# Tunable Nanoparticles with Aggregation‐Induced Emission Heater for Precise Synergistic Photothermal and Thermodynamic Oral Cancer Therapy of Patient‐Derived Tumor Xenograft

**DOI:** 10.1002/advs.202205780

**Published:** 2023-04-20

**Authors:** Leitao Zhang, Chengyan Chu, Xuefeng Lin, Rui Sun, Zibo Li, Sijia Chen, Yinqiao Liu, Jian Wu, Zhiqiang Yu, Xiqiang Liu

**Affiliations:** ^1^ Department of Oral and Maxillofacial Surgery Nanfang Hospital Southern Medical University Guangzhou 510515 China; ^2^ Pingshan District People's Hospital of Shenzhen Pingshan General Hospital of Southern Medical University Shenzhen Guangdong 518118 China; ^3^ Department of Laboratory Medicine Dongguan Institute of Clinical Cancer Research Affiliated Dongguan Hospital Southern Medical University Dongguan 523018 China; ^4^ Center of Hepato‐Pancreato‐Biliary Surgery The First Affiliated Hospital Sun Yat‐sen University Guangzhou Guangdong Province 510080 China; ^5^ State Key Laboratory of Pharmaceutical Biotechnology, School of Life Sciences Nanjing University Nanjing 210023 China

**Keywords:** aggregation‐induced emission, carbon radicals, photothermal therapy, the second near‐infrared window, thermodynamic therapy

## Abstract

The fluorophores in the second near‐infrared (NIR‐II) biological window (1000 – 1700 nm) show great application prospects in the fields of biology and optical communications. However, both excellent radiative transition and nonradiative transition cannot be achieved simultaneously for the majority of traditional fluorophores. Herein, tunable nanoparticles formulated with aggregation‐induced emission (AIE) heater are developed rationally. The system can be implemented via the development of an ideal synergistic system that can not only produce photothermal from nonspecific triggers but also trigger carbon radical release. Once accumulating in tumors and subsequently being irradiated with 808 nm laser, the nanoparticles (NMB@NPs) encapsulated with NMDPA‐MT‐BBTD (NMB) are splitted due to the photothermal effect of NMB, leading to the decomposition of azo bonds in the nanoparticle matrix to generate carbon radical. Accompanied by second near‐infrared (NIR‐II) window emission from the NMB, fluorescence image‐guided thermodynamic therapy (TDT) and photothermal therapy (PTT) which significantly inhibited the growth of oral cancer and negligible systemic toxicity is achieved synergistically. Taken together, this AIE luminogens‐based synergistic photothermal‐thermodynamic strategy brings a new insight into the design of superior versatile fluorescent NPs for precise biomedical applications and holds great promise to enhance the therapeutic efficacy of cancer therapy.

## Graphical abstract

Tunable nanoparticles formulated with aggregation‐induced emission (AIE) heater which cannot only produce photothermal from nonspecific triggers but also trigger carbon radical release is developed. Accompanied by second near‐infrared (NIR‐II) window emission from the NMB, fluorescence image‐guided thermodynamic therapy (TDT) and photothermal therapy (PTT) which significantly inhibited the growth of oral cancer and negligible systemic toxicity is achieved synergistically.

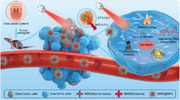

## 1 Introduction

Fluorescence imaging (FLI) as a powerful tool is widely employed for clinical decision‐making and treatment development during endoscopy or surgery, which not only simplifies accurate track drug and direct visualization of tumor but also can furnish spatiotemporal control and noninvasive treatment as well.^[^ ^1^, ^2^ ^]^ Compared to other imaging modalities like magnetic resonance imaging, computed tomography (CT), and positron emission tomography, fluorescence imaging has the characteristics of high spatial and temporal resolution, low cost, non‐invasiveness and good biosafety.^[^ ^3^, ^4^, ^5^, ^6^ ^]^ Under light excitation, the fluorescence emission can be produced by radiative transition of fluorophore.^[^ ^7^, ^8^ ^]^ At the same time, the photothermal effect can also be generated by nonradiative transitions.^[^ ^9^, ^10^ ^]^ In general, the energy may not be interconverted without loss of either of the two pathways in a single system. Therefore, it is definitely appealing and significantly challenging to design clinically practical and valuable fluorophores that can simultaneously with high performance both in radiative transition and nonradiative transition. On the other hand, fluorescence imaging requires a certain wavelength of fluorophore for which blue and/or green excitation light are just compatible with surface imaging applications and yellow or red light (at ≈600 nm) leads to excessive autofluorescence of biosubstrates.^[^ ^11^, ^12^ ^]^ Conventional fluorophores have the intrinsic obstacles in terms of both penetration depth and spatial resolution.^[^ ^13^, ^14^ ^]^ The fluorophore emission wavelength at the second near‐infrared window (NIR‐II;1000–1700 nm) is expected to be the best choice for deep tissue imaging in vivo according to the Stokes shift. ^3b, 5^ Tremendous efforts have been contributed to explore many NIR‐II fluorophores, but the current situation is always confronted with some biosafety limitations, such as poor water solubility and the accompanying fluorescence quenching because of the aggregation in aqueous medium.^[^ ^15^, ^16^ ^]^ Aggregation‐caused fluorescence quenching (ACQ) is a typical problem for achieving effective therapy of fluorophores, and is caused by their low hydrophilicity in physiological environments.^[^ ^17^, ^18^ ^]^ Given the circumstances, aggregation‐induced emission (AIE) is now in the ascendant and its developing foreground is vast which provides a new design viewpoint and direction for the progress of NIR‐II fluorescence imaging.^[^ ^19^, ^20^, ^21^, ^22^ ^]^ Unlike ACQ fluorophores, AIE luminogens are weakly emissive or nonemissive in the solution state but show significantly enhanced fluorescence in the aggregation state. Besides, fluorescence intensity can be increased by subtly designing structures that prevent close intermolecular interactions.^[^ ^23^, ^24^, ^25^ ^]^ In this way, integrating NIR‐II emission into fluorophores with AIE characteristics can probably overcome the above‐mentioned shortcomings and maintain an equilibrium in the balance of radiative and nonradiative energy dissipations through versatile strategies effectively.^[^ ^21^, ^26^, ^27^, ^28^ ^]^

In the past decade, near‐infrared light (NIR)‐sensitive photothermal agents (PTAs) are in high repute worldwide for cancer photothermal therapy (PTT) and the combination treatments.^[^ ^29^ ^]^ However, as soon as irradiation of the NIR‐I biowindow (700–900 nm) PTAs below 0.33 W cm^−2^ threshold, the PTT efficiency can confidently change.^[^ ^30^ ^]^ Moreover, a temperature elevation below 45 °C does not have an ideal antitumor effect and may even produce heat shock proteins (HSP) while above 60 °C may cause deteriorate cancer therapy like inflammation and tumor metastasis.^[^ ^31^, ^32^ ^]^ The solution to this problem lies in the key attributes of PTAs including NIR‐I and/or NIR‐II absorbance and photothermal conversion efficiency. Studies have shown that aggregation‐induced emission luminogens (AIEgens) are potentially applicable in photothermal therapy.^[^ ^33^ ^]^ Surpassingly sparkles will crash out in the unceasing integration of AIEgens‐based NIR‐II FLI and PTT.

One therapeutic modality dependent on free radicals has been verified to be promising for cancer therapy.^[^ ^34^, ^35^, ^36^ ^]^ While the tumor microenvironment (TME) is hypoxic that limits the therapeutic effects of oxygen level‐dependent treatment approaches.^[^ ^37^, ^38^ ^]^ Fortunately, thermodynamic therapy (TDT) as another therapeutic modality has recently been exploited which leveraging oxygen‐independent radicals like carbon radicals to kill tumors substitutes for reactive oxygen species (ROS).^[^ ^39^, ^40^, ^41^ ^]^ Carbon radicals are generated from azo compounds upon triggering under thermal or irradiation stimulation, which are not only used in free radical polymerization, but also applied in biological systems to induce oxidative stress.^15b, 16^ However, the physiological temperature is inefficient to promote initiator cleavage and free radical release to induce the apoptosis of tumor cells. Moreover, 2,2‐azobis[2‐(2‐imidazolin‐2‐yl)propane] dihydrochloride (AIBI) a widely controversial initiator with poor biocompatibility and instability.^[^ ^42^ ^]^ Therefore, it is highly desirable that introducing an in situ nano‐heater with good biocompatibility and stability to accelerate the release of oxygen‐independent cytotoxic free radicals in tumors.

**Scheme 1:**
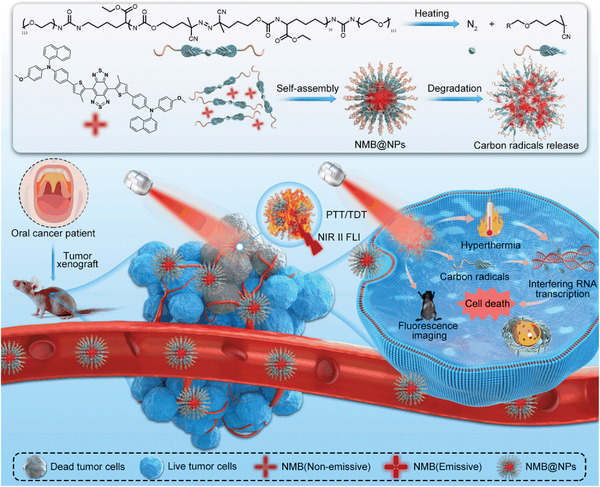
Schematic illustration of the formulation and therapeutic mechanism of NMB@NPs for precise synergistic photothermal and thermodynamic oral cancer therapy of patient‐derived tumor xenograft.

In this report, an AIEgen with NIR‐II FLI guided PTT/TDT (**Scheme** **1**) system has been constructed, wherein azo compounds‐containing nanoparticles (NPs) are facilely fabricated from one‐pot copolymerization of carbon radicals monomer, ethyl 2,6‐diisocyanatohexanoate and poly(ethylene glycol) (PEG) molecules and the AIEgen as a molecular heater was loaded in the hydrophobic core (NMB@NPs). In more detail, NMB@NPs featured with AIE were formulated by the polymers to enhance the water dispersity. Further, the NMB could act as a photothermal agent for enhancing the temperature upon in situ light irradiation. Upon NIR laser irradiation, the heating of NMB@NPs would trigger the NPs to be rapidly decomposed to generate carbon radicals. Importantly, once this delivery system entered the tumor region, NMB@NPs nanomedicine could enable the fluorescent monitoring over the biodistribution and simultaneously inhibit the proliferation of patient‐derived tumor cells by synergetic heat production and carbon radicals release in patient‐derived xenograft (PDX) models (Scheme 1).

## 2 Results and Discussion

### 2.1 Synthesis and Photophysical Property of NMB@NPs

**Figure 1:**
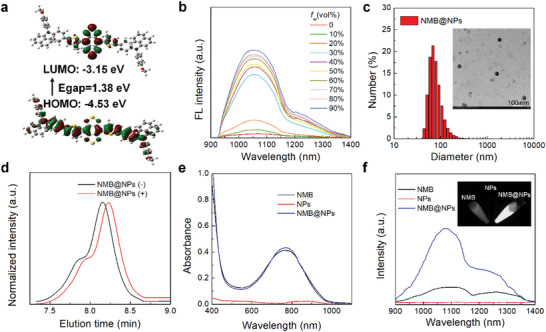
Characterization of NMB and NMB@NPs. a) The HOMO/LUMO gaps of NMB are calculated by density functional theory (DFT). b) FL spectra of NMB in the mixture of DMF and H_2_O with different water fractions (*f*_w_). c) DLS profile and TEM image (inset) of NMB@NPs. d) DMF GPC traces were recorded for the freeze‐drying sample of NMB@NPs before and after the 808 nm laser irradiation for 10 min. e) Absorption spectra of NMB, NPs, and NMB@NPs. f) Fluorescent emission spectra of NMB (*f*_w_ = 30%), NPs, and NMB@NPs, respectively.

The processing and the chemical structure of NMDPA‐MT‐BBTD (NMB) was shown in Figure S1, Supporting Information. 4,8‐dibromo‐1H,5H‐benzo[1,2‐c:4,5‐c“]bis([1,2,5] thiadiazole) chose to be an acceptor and came from commercialization, but the stronger electron donor compound 4 undergoes a series of classic chemical reactions. Related precursor compounds of the NMB were confirmed by NMR spectroscopy and MALDI‐TOF‐MS analysis (Figure S2, Supporting Information). The energy bandgap of NMB was calculated at 1.38 eV (**Figure** **1**a) by the typical electron distribution of the highest occupied molecular orbital and lowest unoccupied molecular orbital to study the NIR‐II‐emitting properties of nanoparticles.^[^ ^43^, ^44^ ^]^ The little bandgap was closely related to the electron donor effect of the arylamine unit and matched very well with the longer emission wavelength of NMB.^[^ ^45^ ^]^ Generally, the widely‐accepted view is that the narrow bandgap is due to the strong intermolecular charge transfer between the electron donor and acceptor units. Moreover, the substituted alkyl chain caused backbone distortion and rotor twisting thus would assist in preventing close packing and reducing intermolecular interactions to improve fluorescence intensity and retain AIE features.^[^ ^46^ ^]^ Fluorescence emission spectra of mixtures with different proportions of DMF and water were obtained to study the AIE property of NMB. As shown in Figure 1b, the NMB was almost non‐emissive in DMF but enhanced fluorescence after being introduced in a mixture of DMF/water with 30%. The thermolabile polymers were fabricated from one‐pot polymerization of three kinds monomers, (E)‐2,2”‐(diazene‐1,2‐diyl)bis(5‐hydroxy‐2‐methylpentanenitrile), ethyl 2,6‐diisocyanatohexanoate, and PEG_5000_‐NH_2_ to afford the resulting thermolabile products (Figure S1, Supporting Information). The chemical structure of the thermolabile polymers was characterized by ^1^H NMR spectrum (Figure S4, Supporting Information). The amphiphilicity was demonstrated by encapsulating a hydrophobic dye Nile red, and the value of low critical micelle concentration was calculated to be ≈65.7 µg mL^−1^ (Figure S5, Supporting Information). Likewise, hydrophobic AIE photothermal dye NMB could directly be encapsulated in the amphiphilic thermolabile polymers, namely NMB@NPs. Furthermore, FT‐IR spectra of NMB and NMB@NPs both showed the characteristic peaks at 650.01, 1502.55, and 1600.92 cm^−1^ corresponding to the C–H stretching vibration of aromatic rings of NMB (Figure S6, Supporting Information), suggesting that NMB was successfully encapsulated in the polymer nanoparticles. As demonstrated in the dynamic light scattering and transmission electron microscopy images, the NMB@NPs and the control polymeric NPs were spherical, and aqueous hydrodynamic diameter was observed to be ≈70 and ≈25 nm, respectively (Figure 1c and Figure S7, Supporting Information). The diameter change was minimal even upon incubation for 7 days, indicating exceptional stability for NMB@NPs at both physiological and simulated culture conditions. Moreover, upon laser irradiation, NMB@NPs were observed to gradually grow larger, expand and cleave, which presumably resulted from the cleavage of thermolabile azo bonds in the polymer backbone, and the polymer degradation was also characterized by GPC traces (Figure 1d and Figure S8, Supporting Information).^[^ ^47^ ^]^ The characteristic absorption and fluorescence emission wavelengths of NMB@NPs were then measured. UV−vis spectrum of NMB@NPs showed stronger absorption at 600−1000 nm (Figure 1e), indicating the successful encapsulation of NMB due to its broad absorption in the range of 600−1000 nm. Moreover, the stability of NMB@NPs was examined in PBS buffer by UV‐vis (Figure S9, Supporting Information). The results show that the UV absorption did not change significantly in 7 days, indicating that NMB@NPs have excellent long‐term stability in aqueous solutions. In addition, the absorption of NMB at varied concentrations was measured (Figure S10, Supporting Information). The content of NMB in NMB@NPs (0.3 mg mL^−1^) was calculated to be 25.4 µg mL^−1^ NMB through the standard curve. Upon photoexcitation at 808 nm, fluorescence from NMB@NPs was clearly observed (Figure 1f), and it exhibited a maximum emission peak at 1090 nm with a broad emission (900–1400 nm) covering NIR‐II region (>1000 nm), which made it have great potential as an imaging agent. To further study the kinetics of thermal responsiveness of NPs, the fluorescence spectra of Nile red@NPs were measured at different temperatures. The results showed that the fluorescence intensity of Nile red@NPs decreased so much over time in higher temperatures, indicating pyrolyzed dissociation of the nanoparticles (Figure S11, Supporting Information). Taken together, the above results demonstrated that NMB@NPs could be decomposed by the photothermal effect‐induced heat production.

### 2.2 Photothermal Properties of NMB@NPs

**Figure 2:**
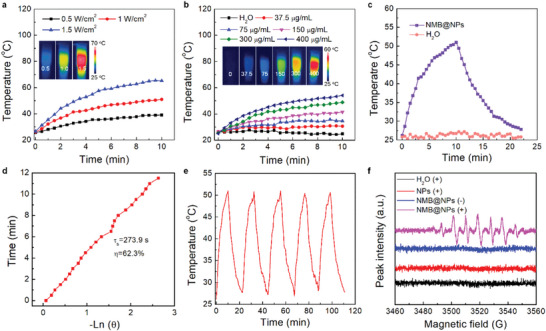
Photothermal properties of NMB@NPs. a) Photothermal heating curves and infrared thermal images of NMB@NPs (0.3 mg mL^−1^) upon laser irradiation with diverse power densities and durations. b) Photothermal heating curves and infrared thermal images of NMB@NPs at various concentrations under 808 nm laser irradiation (1.0 W cm^−2^) for 10 min. c) Photothermal effect of NMB@NPs in water with 808 nm laser irradiation d) Time constant for heat transfer from the system is determined to be *τ*_s_ = 273.9 s by applying the linear time data from the cooling period (after 600 s) versus negative natural logarithm of temperature. e) Photothermal stability evaluation of NMB@NPs solution f) ESR spectra of POBN in H_2_O, NPs, and NMB@NPs solution under 808 nm laser irradiation for 10 min.

In view of the outstanding NIR‐I absorption of NMB, which made NMB@NPs be fit for tumor PTT, the photothermal property of NMB@NPs in water were systematically evaluated. As expected, the temperature of the NMB@NPs dispersion could be effectively increased (Figure S12 and S13, Supporting Information). With increasing the concentration from 0 to 400 µg mL^−1^ and the power density of laser, the temperature changes of the solution could rapidly increase from 25.0 to 54.1 °C and 26.7 to 65.2 °C within 10 min, respectively (**Figure** **2**a,b). And the photothermal conversion efficiency (*η*) of NMB@NPs at 808 nm via the standard detection method was calculated to be 62.3% which was much higher than some photothermal agents reported (Figure 2c,d).^[^ ^48^, ^49^, ^50^ ^]^ After 10 min irradiation with an 808 nm laser then turned off the laser to cool down naturally. Repeating the above operation for five on‐and‐off cycles indicated no appreciable reduction of temperature rise trend of NMB@NPs, suggesting superior photothermal stability for NMB@NPs (Figure 2e). At the same time, NMB exhibited better photostability which compared with the commercial dye ICG (Figure S14, Supporting Information). All these results indicated that NMB@NPs had excellent photothermal activity, which could trigger the NPs to be efficiently decomposed to generate carbon radicals.

In the following, the carbon radical generation ability of NMB@NPs was investigated using the electron spin resonance (ESR) technique. The released reactive free radicals were captured by the spin trap a‐(4‐Pyridyl N‐oxide)‐N‐tert‐butylnitrone (POBN). As shown in Figure 2f, typical alkyl radical signals were observed from NMB@NPs upon 808 nm laser irradiation using POBN. In contrast, no ESR radical signal was found in the H_2_O and NPs treated groups in the absence of laser irradiation. Moreover, the same concentration of NMB@NPs did not observe ESR signals without the laser irradiation. These results demonstrated that the carbon radical generation capability of NMB@NPs was put down to the heating of AIE upon laser irradiation. Then the heat‐triggered generation of carbon radicals was investigated by 1,3‐diphenylisobenzofuran (DPBF), which was oxidized by the radicals, exhibiting typical characteristic absorbance reduction at ≈410 nm. As shown in Figure S15, Supporting Information, the time‐dependent generation of carbon radicals was observed when DPBF was incubated with NPs at 37, 45, and 50 °C, respectively. As a comparison, the absorption of DPBF alone dissolved in DMF showed little change in the absence of NPs at the same condition. In addition, 2,2’‐azino‐bis (3‐ethylbenzothiazoline‐6‐sulfonic acid) (ABTS) (one of the most common free radical capture reagents) was employed to prove the generation of free radicals by NPs with thermal stimulation. As shown in Figure S16, Supporting Information, a characteristic absorbance peak at 300–850 nm gradually increased especially in a higher temperature of 50 °C, indicating the decomposition of NPs and release of free radicals.

### 2.3 In Vitro Cell Uptake and Intracellular Free‐Radical Detection

**Figure 3:**
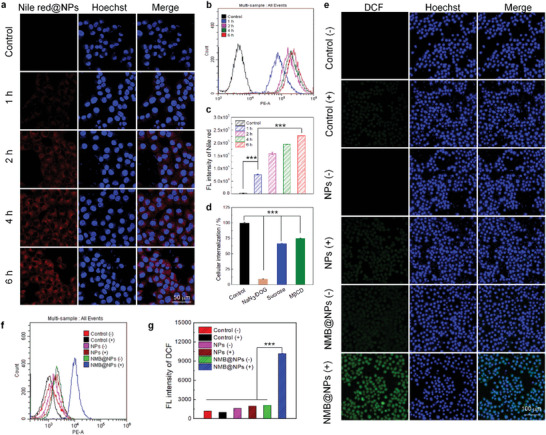
Cellular uptake of Nile red@NPs against Cal‐27 cells and the measurements of heat‐controlled generation of carbon radicals. a) Confocal images of Cal‐27 cells after incubation of the cells with Nile red@NPs (0.3 mg mL^−1^) for different times. The scale bar is 50 µm. (*P* values: ***P < 0.001) b) Flow cytometry analysis of Cal‐27 cells upon different treatments with Nile red@NPs. c) Statistical analysis of the data in (b). d) Flow cytometry analysis of Cal‐27 cells toward Nile red@NPs (0.3 mg mL^−1^) in the presence of different endocytosis inhibitors. e) CLSM images for Cal‐27 cells staining with DCFH‐DA (a universal indicator of oxidative stress fluorescent probe, blue) with different treatments. f) Flow cytometry fluorescence histogram of DCF intensity in Cal‐27 cells with different treatments. g) Statistical analysis of the data in (f) (*P* values: ***P < 0.001).

The decreased cytotoxicity of Cal‐27 cells to NMB@NPs drugs was related to the reduced endocytosis. Therefore, the cellular uptake of NMB@NPs was investigated towards Cal‐27 tumor cells by confocal laser scanning microscopy (CLSM) imaging and flow cytometry analysis. As already mentioned, we used Nile red (Nile red@NPs) instead of NMB molecule to study intracellular uptake characteristics. The cellular fluorescence intensities became stronger and spread over cytoplasm as the incubation time increased (**Figure** **3**a), revealing Nile red@NPs can be effectively internalized by the Cal‐27 cells. In addition, as illustrated in Figure 3b,c, more Nile red@NPs were internalized as the incubation time prolonged from 1 to 6 h, which agreed well with the CLSM results. These data indicated that NMB@NPs could be internalized, which was highly favorable for causing subsequent cytotoxicity. Inspired by the efficient cellular uptake of Nile red@NPs, further evaluation the endocytic pathways of Nile red@NPs using various pathway inhibitors was performed by flow cytometry analysis (Figure S17, 3d). In contrast with control group, remarkable decreasing in the fluorescence signal of Nile red@NPs was visualized after incubation with NaN_3_/DOG (0.1% NaN_3_, 50 mM DOG), sucrose (225 mM) and methyl‐*β*‐cyclodextrin (M*β*CD) (5 mM) in Cal‐27 cells, respectively. Because the cellular endocytosis procedure was energy‐dependent and could be inhibited by NaN_3_/DOG (ATP depletion agent), sucrose (a clathrin‐mediated endocytosis inhibitor), and M*β*CD (a caveolae‐mediated endocytosis inhibitor).^[^ ^51^, ^52^, ^53^ ^]^ Furthermore, the inhibitory effect of caveolae‐mediated endocytosis inhibitor was more significant than that of clathrin‐mediated endocytosis inhibitor. These results further indicated that the energy‐dependent pathway played a vital role in the internalization of NMB@NPs and the caveolae‐mediated endocytosis model was predominant for cell staining by the NMB@NPs.

Subsequently, the carbon radical generation in Cal‐27 cells upon treating with NMB@NPs after irradiation was investigated using 2’,7’‐dichlorodihydrofluorescein diacetate (DCFH‐DA), a nonfluorescent molecule that can passively diffuse into cells and be oxidized to produce a bright green fluorescence compound DCF. Clear green fluorescence of DCF was observed in cells after incubating with NMB@NPs followed by light irradiation, while the weakest fluorescence signal was observed without laser‐irradiation (Figure 3e). The control group not treated with any NMB@NPs was also negligible fluorescence signal with/without laser‐irradiation. Flow cytometry analysis further revealed that the better performance of carbon‐free radical production ability with NMB@NPs (Figure 3f,g), which was consistent with the observation by confocal microscopy imaging. These findings indicated that NMB@NPs could readily release carbon radicals under the laser irradiation.

### 2.4 In Vitro Anti‐Cancer Activity of the NMB@NPs

**Figure 4:**
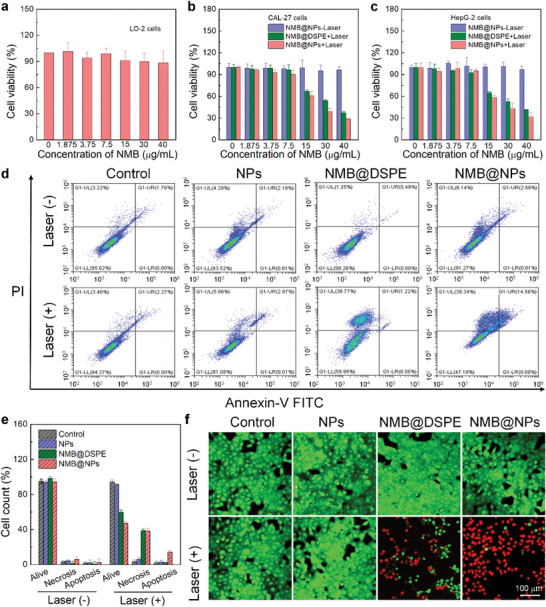
In vitro anti‐cancer activity of the NMB@NPs. a) Relative cellular viabilities of LO‐2 cells treated with NMB@NPs at various concentrations upon incubation for 36 h. Cell viability of b) Cal‐27 cells and c) HepG‐2 cells after incubating with different NMB@NPs concentrations before and after irradiation by MTT assay. d) Cell apoptosis assays for Cal‐27 cells treated with after various treatments before and after laser irradiation. e) Statistical analysis for the percentage of apoptosis and necrosis in (d). f) Live/dead cell staining images of Cal‐27 cells after different treatments.

In vitro synergistic antitumor efficacy of PTT and TDT was evaluated by treatment of NMB@NPs following NIR irradiation. NMB@NPs in the dark showed minimal cytotoxicity towards normal cells (LO‐2) even if the concentration was up to 40 µg mL^−1^ (NMB) after 36 h incubation, indicating the negligible cytotoxicity of NMB@NPs (**Figure** **4**a). Furthermore, upon laser irradiation (808 nm, 1.0 W cm^−2^) for 10 min, the cells incubated with low concentration of NMB@NPs showed no significant decrease in viability, suggesting the negligible cytotoxicity at the studied power density (Figure 4b). In striking contrast to the above results, after 36 h treatment with NMB@NPs at the stated concentrations under irradiation, the viabilities of Cal‐27 were only 30%. Compared with the single PTT (NMB@DSPE), the combined treatment by NMB@NPs (+Laser) was much effective in killing cancer cells. In addition, the therapeutic effects of NMB@NPs in HepG‐2 cells were evaluated, indicating the broad anticancer potency of NMB@NPs (Figure 4c). The combination therapeutic efficacy could be further enhanced along with the carbon radical generation. The feasibility of NMB@NPs in proliferation inhibition towards Cal‐27 cells in vitro was further confirmed by Annexin V‐fluoresceine isothiocyanate/propidium iodide (Annexin V‐FITC/PI) staining assays. With 808 nm laser irradiation, the NMB@NPs group was more effective at inducing apoptosis than NPs and NMB@DSPE (Figure 4d,e). Early apoptosis and late apoptosis at below 10% could be detected for the samples in the absence of laser irradiation or in PBS‐treated cells in the presence of laser irradiation. The result of NMB@NPs with laser irradiation further confirmed that the integration of photothermal effect with generated carbon radicals could efficiently induce cancer‐cell apoptosis. In order to visually evaluate this great antiproliferation effect, the cells were stained with calcein‐AM and propidium iodide (PI) to identify live and dead cells, respectively (Figure 4f). Cells in the control group (±Laser) and NPs, NMB@DSPE (‐Laser), NMB@NPs (‐Laser) treatment groups all displayed green FL, which suggested that pure laser irradiation or NMB@NPs alone could not kill cancer cells. The results demonstrated the excellent biocompatibility of NMB@NPs in the dark. While after NIR laser irradiation, bright red fluorescence evidently appeared in the groups treated with NMB@NPs, both green and red fluorescence was detected in NMB@DSPE, which reveals that a combination of photothermal effect and generated free radicals are efficient in killing Cal‐27 cells.

**Figure 5:**
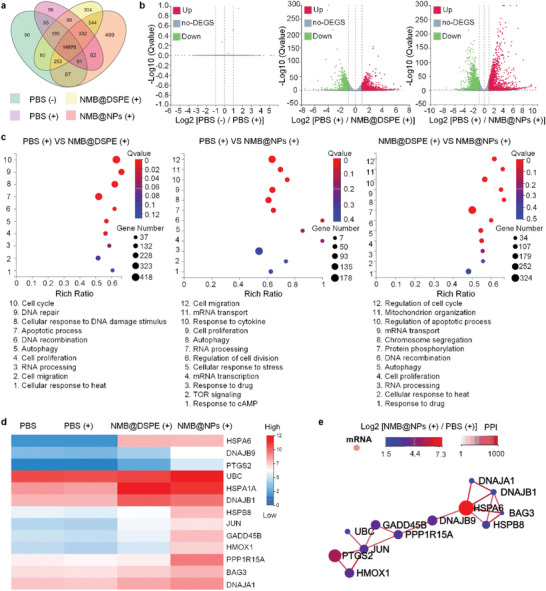
RNA sequencing of Cal‐27 cells after different treatments. a) The VENN graph represented gene expression relationship between each group. b) Differential gene volcano map represented the number of up‐regulated or down‐regulated gene expressions between PBS and different nanoparticle groups. c) The enrichment analysis of Gene Ontology (GO) between different samples. d) Differential gene clustering heat map of different samples. e) The graph of protein–protein interaction (PPI) network compared with PBS (+) and NMB@NPs (+) group.

To further explore the underlying biological mechanism of synergistic tumor therapy of NMB@NPs, we performed whole genome RNA expression sequencing (RNAseq) on Cal‐27 cells exposed to various treatments. By RNA sequencing, tumor gene expression profiles of PBS (+Laser), NMB@DSPE (+Laser), and NMB@NPs (+Laser) groups were revealed and were compared with PBS (‐Laser) group, which was considered as a control. Following with bioinformatic analysis, consistency analysis indicated a high consistency in gene expression of each group, except for a distinct difference shown in NMB@NPs (+Laser), which was deduced to be caused by the photothermal effect of NMB. The gene expression relationship between each group was displayed in the VENN graph (**Figure** **5**a). Among the 17 755 examined genes, there were 90, 98, 304, and 499 gene transcripts uniquely upregulated in Cal‐27 cells treated by PBS (‐Laser), PBS (+Laser), NMB@DSPE (+Laser), and NMB@NPs (+Laser), respectively. Most genes that exclusively expressed in NMB@NPs (+Laser) were attributed to the cooperation of photothermal therapy and carbon radicals respectively. For ensuring differential expression level in response to different treatments, Deseq2 was utilized to calculate the relative transcript level compared to the PBS group. The hierarchical clustering of these altered expression gene sets was revealed with absolute log2 fold‐change ≥ 1 and *p* < 0.05 as a filter. As shown in the volcano plots (Figure 5b), there were 4357 genes were upregulated while 4768 genes were downregulated in NMB@NPs (+Laser) group, which were much more significant of gene expression than that of PBS (+Laser) (0 genes and 0 genes), and NMB@DSPE (+Laser) (4015 genes and 3978 genes). Furthermore, relative pathways were analyzed with the “biological process” in the Kyoto Encyclopedia of Genes and Genomes and the supervised gene ontology (GO) database. Based on Figure 5c, multiple signaling pathways were significantly affected, e.g., signal transduction, RNA processing, cell proliferation, and apoptotic process. Compare to the control group, NMB@NPs (+Laser) significantly affected cell migration, cell proliferation, and mRNA transcription thanks to the best photothermal and alkyl radical generation effects. Many of these dysregulated genes encoded heat shock proteins (HSP) and co‐chaperones for HSPs such as HSPA6, HSPB8, HMOX1(HSP32), DNAJB1 (HSP40 member B1), DNAJB9 (HSP40 member B9), DNAJA1 (HSP40 member A1), and BAG3 (Figure 5d). In addition, transcriptional alteration of some apoptosis genes (GADD45B, PPP1R15A, and JUN) indicated that NMB‐NP+L effectively promoted the apoptosis of cancer cells. The graph of protein–protein interaction (PPI) network showed HSP‐related genes of NMB@NPs (+Laser) group such as HMOX1, HSPA6, PPP1R15A, and DNAJB9 were up‐regulated compared to PBS group, which proved that NMB@NPs (+Laser) can induce photothermal therapy effectively (Figure 5e). All in all, NMB@NPs (+Laser) treatment integrated drug‐based cytotoxicity with photothermal effects and carbon radicals to construct a comprehensive and highly efficient therapeutic system for tumor therapy.

### 2.5 In Vivo NIR‐II Imaging and Photothermal Imaging

**Figure 6:**
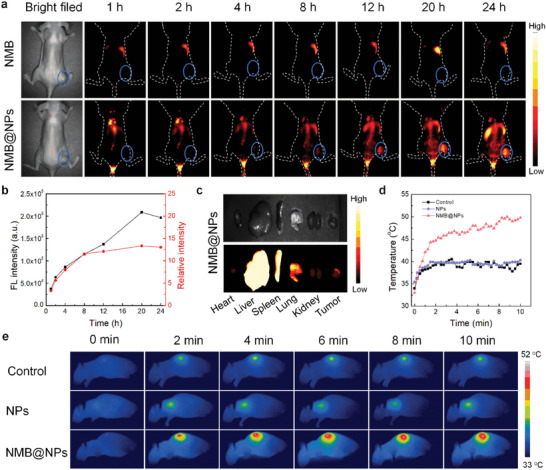
In vivo fluorescent/thermal imaging and biodistribution of NMB@NPs. a) In vivo NIR‐II FL imaging results of free NMB and NMB@NPs at different time intervals. b) Fluorescent signal changes in the tumor sites of mice injected with NMB@NPs. c) Ex vivo NIR‐II FL images of major organs and tumors after injection with NMB@NPs at 24 h. d) Temperature evolution profile in tumors of mice with different groups at varied periods. e) Real‐time infrared thermal images of oral cancer xenograft model mice.

In vivo tumor penetration and imaged‐guiding capability of NMB@NPs were further investigated through, oral cancer PDX model. Representative NIR‐II fluorescence images at a specific time point (0, 1, 2, 4, 8, 12, 20, and 24 h) were acquired with a filter of 1100 nm (**Figure** **6**a,b). As the extension of observation time, the legible and strong fluorescence signal with excellent signal‐to‐noise ratio was acquired for NMB@NPs inside the tumor and reached a peak at 20 h postinjection, thus it suggested that 20 h would be the optimal time point of light illumination for the subsequent PTT. In contrast, the fluorescence of tumor treated with NMB could be hardly visualized. Accompanied by normal physiological metabolism 24 h later, the imaging signal diminished. The imaging results indicated that NMB@NPs could efficiently accumulate at the tumor site and follow‐up the therapeutic process in real time. To evaluate the biodistribution of NMB@NPs, the tumor and main organs were collected after 24 h postinjection (Figure 6c). The liver and spleen showed very high fluorescence intensity, and the fluorescence signal in the tumor site was slightly weak, which indicated that NMB@NPs accumulated mainly in the liver and spleen.

Encouraged by the nanoparticles exhibited superior photothermal ability in vitro and excellent tumor accumulation, the temperature change during light irradiation was recorded at the established tumor xenograft site by an IR thermal camera. Mice were injected with PBS, NPs, and NMB@NPs (NMB: 2.0 mg kg^−1^), respectively. The tumors were irradiated by 808 nm laser (1.0 W cm^−2^) for 10 min 20 h after the injection (Figure 6d). The temperature changes for the PBS and NPs groups increased by ≈5.3 and ≈4.9 °C, respectively. While temperature for the NMB@NPs group increased remarkably by ≈17.0 °C in 10 min and increased to 50.0 °C with the same parameters, which was high enough to cause apoptosis of oral cancer cells by destroying the cell membrane, damaging the cytoskeleton and inhibiting DNA synthesis, etc.^[^ ^54^, ^55^ ^]^

### 2.6 Anti‐Tumor Efficacy of NMB@NPs in a PDX Mouse Model

**Figure 7:**
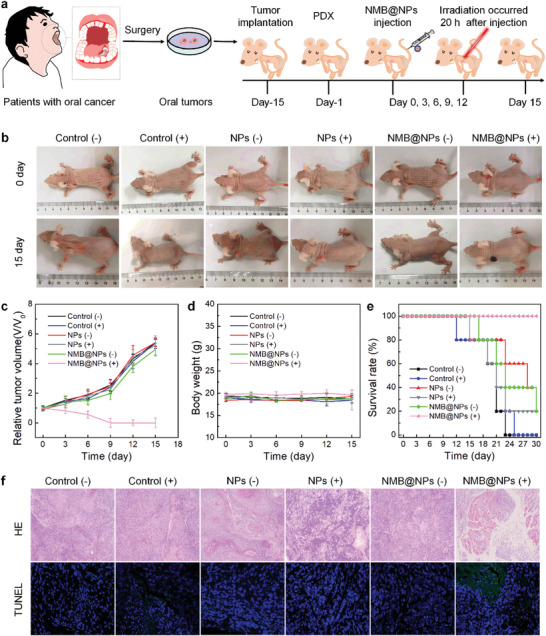
Evaluation of antitumor efficacy of NMB@NPs in the subcutaneous PDX mouse model. a) Treatment regimen of the subcutaneous PDX in BABL/c nude mice. b) Digital images of representative mice at day 0 and day 15. c) Tumor growth curves until day 15. (*P* values: ***P < 0.001). d) Body‐weight changes in different groups. e) Survival curves of mice in different treatment groups. f) Typical images of H&E and TUNEL (20x) stained tumor tissues of oral tumor‐bearing mice after various treatments.

Finally, we continued to apply NMB@NPs for in vivo cancer therapy of oral cancer PDX model (**Figure** **7**a). The tumor‐bearing BALB/c mice were randomly divided into six groups (*n* = 5): (1) PBS (‐Laser), (2) PBS (+Laser), (3) NPs (‐Laser), (4) NPs (+Laser), (5) NMB@NPs (‐Laser), (5) NMB@NPs (+Laser). The mice were intravenously injected with NPs or NMB@NPs at a fixed NPs dose of 20 mg kg^−1^, respectively. At 20 h post‐injection, the 808 nm laser irradiation was operated at tumor sites for 10 min at a power density of 1.0 W cm^−2^. As shown in Figure 7b, c, the single employment of NPs or 808 nm laser irradiation could not inhibit tumor growth. The administration with NMB@NPs without irradiation also showed no significant inhibition. Comparatively, the tumors in mice treated with NMB@NPs plus laser irradiation were gradually suppressed until completely eliminated during treatment, which fully revealed the synergistic PTT/TDT treatment effect of NMB@NPs. The mean body weight of mice did not change obviously in all the groups within the whole treatment period (Figure 7d). Significantly, the mice's survival time was obviously prolonged, similar to those of healthy mice (Figure 7e). Next, we analyzed the amounts of dead cells and the corruption extent of extracellular matrix in the tumor tissue to further obtain insight into the antitumor effect of different treatments by hematoxylin‐eosin (H&E) staining and TUNEL assay. Likewise, the group of NMB@NPs plus NIR could trigger the tumor cell apoptosis and exhibited apparently better antitumor efficacy on the oral cancer PDX model (Figure 7f).

At last, the biocompatibility of NMB@NPs was explored by HE, hemolysis analysis, blood routine examination, and biochemical analysis. The major organs were harvested on the 15th day for the histological examination using the H&E staining technique. Figure S19, Supporting Information showed the major organs with no apparent pathological abnormality in the six groups, indicating the low in vivo systemic toxicity of NMB@NPs. Lower hemolysis (<5%) was observed when mouse erythrocytes were treated with diverse samples of NMB@NPs (Figure S20, Supporting Information). In addition, the mice treated with PBS or NMB@NPs did not clearly differ in the significant index of the blood hematology and biochemistry (Figures S21 and S22, Supporting Information). All the above results proved the good biocompatibility of NMB@NPs, which possess the remarkable potential in heat‐controlled carbon radical generation as well as cancer diagnosis and treatment.

## 3 Conclusion

In this work, we have successfully demonstrated a PTT/TDT drug therapeutic nanomedicine platform by encapsulating AIEgens within the azo‐containing polymer to achieve more efficient antitumor efficacy. Upon exposure to 808 nm laser irradiation, NMB@NPs better controls the balance between fluorescence and photothermal effect. The AIEgens‐based NIR‐II fluorescence imaging can efficiently localize tumor sites and real‐time monitor the therapeutic process, showing high detection sensitivity, resolution, and deep imaging depth. The photothermal effect of NMB@NPs and carbon radical released by thermal trigger of azo‐containing polymer show high tumor inhibition rates without serious systemic toxicity on oral cancer PDX mouse model. In summary, the presence of simultaneous diagnostic and therapeutic capabilities in NMB@NPs could open up an exciting new field in personalized precise medicine for oral cancer treatment.

## Conflict of Interest

The authors declare no conflict of interest.

## Supporting information

Supporting Information

## Data Availability

The data that support the findings of this study are available in the supplementary material of this article or from the corresponding authors upon reasonable request.
