# Supplementary material for: Tunable Nanoparticles with Aggregation‐Induced Emission Heater for Precise Synergistic Photothermal and Thermodynamic Oral Cancer Therapy of Patient‐Derived Tumor Xenograft
Source: Adv Sci (Weinh). 2023 Apr 20;10(17):2205780. doi: 10.1002/advs.202205780 (PMC10265040; doi:10.1002/advs.202205780)
Supplement: Supplementary file 1 — Supporting Information [file ADVS-10-2205780-s001.pdf]

ADVANCED SCIENCE
Open Access

Supporting Information

for *Adv. Sci.*, DOI 10.1002/advs.202205780

Tunable Nanoparticles with Aggregation-Induced Emission Heater for Precise Synergistic Photothermal and Thermodynamic Oral Cancer Therapy of Patient-Derived Tumor Xenograft

Leitao Zhang, Chengyan Chu, Xuefeng Lin, Rui Sun, Zibo Li, Sijia Chen, Yinqiao Liu, Jian Wu, Zhiqiang Yu* and Xiqiang Liu**

Supporting Information

Tunable Nanoparticles with Aggregation-Induced Emission Heater for Precise Synergistic Photothermal and Thermodynamic Oral Cancer Therapy of Patient-Derived Tumor Xenograft

Leitao Zhang, Chengyan Chu, Xuefeng Lin, Rui Sun, Zibo Li, Sijia Chen, Yinqiao Liu, Jian Wu,* Zhiqiang Yu,* Xiqiang Liu*

Experimental Section

Materials.

1-Bromonaphthalene (J&K Chemical Scientific Ltd.), 4-methoxy-aniline (J&K Chemical Scientific Ltd.), 1,4-bromoiodobenzene (J&K Chemical Scientific Ltd.), 3-Methylthiophene (J&K Chemical Scientific Ltd.), palladium diacetate (Energy Chemical Ltd.), 1.1'-Binaphthyl-2.2'-diphemyl phosphine (Energy Chemical Ltd.), cesium carbonate (J&K Chemical Scientific Ltd.), Bis(triphenylphosphine)palladium(II) chloride (J&K Chemical Scientific Ltd.), sodium tert-butoxide (J&K Chemical Scientific Ltd.), 4,5-Bis(diphenylphosphino)-9,9-dimethylxanthene (J&K Chemical Scientific Ltd.), n-butyllithium (J&K Chemical Scientific Ltd.), tributyltin chloride (Energy Chemical Ltd.), 4,8-dibromo-1H,5H-benzo[1,2-c:4,5-c']bis([1,2,5] thiadiazole) (HWRK Chem Ltd.), Dry toluene (J&K Chemical Scientific Ltd.) were analytical reagents. All these materials were used without further purification. Tetrahydrofuran (THF) were purchased from Sinopharm Chemical Reagent Co. Ltd. and distilled before use. Ethyl acetate (EA), Dichloromethane (DCM) and petroleum ether (PE) were obtained from Sinopharm Chemical Reagent Co. Ltd. and used without further purification. DMSO, Diethyl ether, and all other organic solvents used in this study were analytical-grade products from the Guangzhou Chemical Reagent Company, Guangzhou, China. 3-(4,5-Dimethylthiazolyl-2)-2,5-diphenyltetrazolium bromide (MTT), 2-Deoxy-D-

glucose, 2',7'-dichlorfluorescein-diacetate (DCFH-DA), Calcein AM, propidium iodide (PI), Annexin V-fluorescein isothiocyanate/propidium iodide (Annexin V-FITC/PI) and 4',6-diamidino-2-phenylindole (DAPI) were purchased from Sigma-Aldrich Co. (MO, USA). Nile red β-Cyclodextrin were purchased from Mindray, Chemical Technology Co., Ltd. 3-Amino,4-aminomethyl-2,7-difluorescein, diacetate (DAF-FM DA) and Hoechst 33342 were purchased from the Beyotime Institute of Biotechnology. Ltd. Fetal bovine serum (FBS), penicillin, streptomycin, and Dulbecco's modified Eagle's medium (DMEM) were obtained from GIBCO and used as received. Lyso-Tracker was obtained from Thermo Fisher Invitrogen (MA, USA). Water used in this study was deionized with a Milli-QSP reagent water system (Millipore) to a specific resistivity of 18.4 MΩ·cm. The other reagents were used without further purification.

Characterization.

^{1}H and ^{13}C NMR spectra were measured on a Unity-400 NMR spectrometer using $CDCl_3$ as solvent and tetramethylsilane (TMS) as a reference. The UV−vis absorption spectra were performed using a PerkinElmer Lambda 365 spectrophotometer. Mass spectra (MS) were measured with a GCT premier CAB048 mass spectrometer in MALDI-TOF mode. The photoluminescence (PL) spectra were conducted on a Horiba Fluorolog-3 spectrofluorometer. The size distribution and morphology of the particles were determined using a dynamic light scattering (DLS, Malvern Zetasizer Nano-ZS 90, UK) and transmission electron microscopy (TEM, JEOL JEM-2100, Janapa) at an accelerating voltage of 200 kV. UV−vis-NIR extinction spectra were analyzed by a UV−vis spectrophotometer (Shimadzu, UV-2600, Japan). Fluorescence emission spectra were obtained using a fluorescence spectrophotometer (Laser Flash Photolysis Spectrometer, LP 920, UK). The temperature and thermal images were recorded on infrared thermal camera (Fotric, Fotric 322Pro, Shanghai, China). Cellular uptake was observed by a confocal laser scanning microscopy (CLSM, Carl Zeiss LSM 880 META, Germany). Intracellular

uptake, ROS detection and cell apoptosis were examined by Beckman Cytomics FC 500. *In vivo* NIR-II fluorescence imaging was collected with NIR-OPTICS Series III 900/1700 small animal imaging system (Suzhou NIR-Optics Technology Co., Ltd., China). Animal experiment methods were in accordance with the regulations of the Regional Ethics Committee for Animal Experiments approved by the Administrative Committee of Laboratory Animals of Southern Medical University.

Computing methods.

Density functional theory (DFT) calculations were carried out by the B3LYP/6-31G (d, p), Gaussian 09 package. B3LYP/6-31G (d, p) was selected as the basis set method according to the density functional theory (DFT), the optimal molecular structure and the highest occupied molecular orbital (HOMO) energy and lowest unoccupied molecular orbital (LUMO) energy level information of the target product on the CPU workstation were calculated by the Gaussian 09W program.[S1]

Synthesis of compound 2.

Compound 1 was synthesized according to the literature.[S1] To the solution of 3-Methylthiophene (98 mg, 1 mmol) in the 10 mL dry THF, then the mixture was stirred and cooled down to -80 °C under nitrogen. After that, the n-butyl lithium (0.5 mL, 2.4 mM) was added in the mixture and stirred for 2 h. Then the tributyltin chloride (325 mg, 1.0 mmol) was added in the solution and stirred at room temperature for 8 h. Then the mixture was concentrated under reduced pressure. The resultant product was directly used in the next step reaction without purification.

Synthesis of compound 3.

To the solution of compound 1 (404 mg, 1.0 mmol) and compound 2 (387 mg, 1.0 mmol) in 10 mL dry toluene under nitrogen. Then the bis (triphenylphosphine) palladium (II) chloride (36 mg, 0.05 mmol) was added in the mixture and the mixture was stirred and heated to 110 ^{o}C for 8 h. After cooling to room temperature, the mixture was separated and the organic layer was concentrated under reduced pressure. The desired residue was purified on a silica-gel column using petroleum

ether/ethyl acetate (v/v 20:1-5:1) as eluent. A yellow-green solid of compound 3 was obtained in 87 % yield. ^{1}H NMR (400 MHz, Chloroform-*d*) δ 7.95 (d, *J* = 8.2 Hz, 1H), 7.86 (d, *J* = 8.2 Hz, 1H), 7.73 (d, *J* = 8.2 Hz, 1H), 7.44 (t, *J* = 7.7 Hz, 3H), 7.37 – 7.33 (m, 3H), 7.30 (d, *J* = 8.2 Hz, 1H), 7.09 (d, *J* = 8.4 Hz, 2H), 7.00 (s, 1H), 6.85 – 6.71 (m, 5H), 3.76 (s, 3H), 2.61 (q, *J* = 7.5 Hz, 3H).

Synthesis of compound 4.

To the solution of compound 3 (421 mg, 1.0 mmol) in the 20 mL dry THF, then the mixture was stirred and cooled down to -80 °C under nitrogen. After that, the n-butyl lithium (0.5 mL, 2.4 mM) was added in the mixture and stirred for 2 h. Then the tributyltin chloride (325 mg, 1.0 mmol) was added in the solution and stirred at room temperature for 8 h. Then the mixture was concentrated under reduced pressure. The resultant product was directly used in the next step reaction without purification.

Synthesis of compound NMDPA-MT-BBTD.

To the solution of compound 4 (711 mg, 1.0 mmol) and 4,8-dibromo-1H,5H-benzo[1,2-c:4,5-c']bis([1,2,5] thiadiazole) (175 mg, 0.5 mmol) in 10 mL dry toluene under nitrogen. Then the bis (triphenylphosphine) palladium (II) chloride (72 mg, 0.1 mmol) was added in the mixture and the mixture was stirred and heated to 110 °C for 8 h. After cooling to room temperature, the mixture was separated and the organic layer was concentrated under reduced pressure. The desired residue was purified on a silica-gel column using petroleum ether/ethyl acetate (v/v 20:1-2:1) as eluent. A brown solid of compound NMDPA-MT-BBTD was obtained in 52 % yield. ^{1}H NMR (400 MHz, Chloroform-*d*) δ 7.96 (d, *J* = 8.2 Hz, 2H), 7.88 (d, *J* = 8.2 Hz, 2H), 7.76 (d, *J* = 8.2 Hz, 2H), 7.51 – 7.42 (m, 8H), 7.42 – 7.30 (m, 4H), 7.23 (s, 2H), 7.18 – 7.10 (m, 4H), 6.88 – 6.78 (m, 8H), 3.78 (s, 6H), 2.25 (s, 6H). ^{13}C NMR (101 MHz, $CDCl_3$) δ 155.91, 153.03, 149.18, 147.42, 143.43, 141.00, 140.58, 135.32, 131.05, 128.47, 128.37, 126.86, 126.71, 126.44, 126.39, 126.21, 126.03, 125.96, 125.70, 124.33, 119.41, 115.86, 114.73, 55.52, 14.17. MALDI-TOF, *m/z*:

$[M]^+$ calcd. 1032.24, found 1032.45.

Synthesis ofPolymer.

(E)-2,2'-(diazene-1,2-diyl)bis(5-hydroxy-2-methylpentanenitrile) (0.15 mmol, 37.8 mg), and ethyl 2,6-diisocyanatohexanoate (0.16 mmol, 36.2 mg) were suspended in 5 mL anhydrous DMF. After magnetic stirring for 24 h, PEG_{5000}-NH_2 (2 mmol, 100 mg) was added to the reaction mixture. After magnetic stirring for another 24 h, 5 mL mixture was added into 15 mL of deionized water under sonication, followed by dialysis in a dialysis bag (MWCO: 7000 Da). After 72 h, the solution was freeze-dried under reduced pressure to obtained 112 mg white polymer. The polymer was analyzed by ^{1}H NMR.

Critical micelle concentration (CMC) determination.

a Nile Red 2 mM stock solution was prepared in MeOH. Then NPs micelle samples (1 mg/mL) with various volume were incubated with 1 μM of Nile Red in a final volume of 600 μL. The solution was then consecutively the ultrasonic shaking with 15 min followed by 24 h incubation at 37 °C. Fluorescence-emission spectra (excitation 550 nm) were recorded for an emission range between 560 and 800 nm. At concentrations close to critical micelle concentration, it should be observed a sharp increase in fluorescence intensity and a hypsochromic effect.

Calculation of photothermal conversion efficiency.

NMB@NPs, NPs and deionized water were upon 808 nm laser at a power density of 1.0 W/cm^2 for 10 min. The real-time temperature was recorded during this process and lasted until the dispersions returned to initial temperature. The photothermal-conversion efficiency of NMB@NPs was determined according to the previous method. [1-2] Details are as follows:

$$\eta_T = \frac{hS\Delta T_{max} - Qg}{I(1-10^{-A_{\lambda 808}})} \quad \text{(S1)}$$

$$\theta = \frac{T_{surr} - T}{T_{surr} - T_{max}} \quad \text{(S2)}$$

$$t = \tau_s(-\ln\theta) \quad \text{(S3)}$$

$$\tau_s = \frac{m_D C_D}{hA} \tag{S4}$$

Where h is the heat transfer coefficient, S is the surface area of the container, and the value of hS can be obtained from Equation S2, S3, and S4. Where T is the temperature of NMB@NPs, T_{max} is the maximum system temperature and T_{surr} is the initial temperature. I is the laser power and $A_{\lambda 808}$ means the absorbance of nanoparticle at the wavelength of 808 nm. Q_g (0.0251 J/s) is the energy that absorbed by the solvent. Where τ_s is the sample system time constant. m_D and C_D are the mass (1.09 g) and heat capacity (4.2 J/g) of deionized water, respectively.

Cellular uptake.

Cal-27 cells were cultured in DMEM supplemented with 15% (v/v) fetal bovine serum, 1% (v/v) penicillin, and 1% (v/v) streptomycin, respectively. Cells were incubated in a humidified incubator at 37 °C with 5% CO_2. Cancer cells were seeded on a glass bottom petri dish for 12 h before diverse treatments and then examined with CLSM imaging. Hoechst 33342 and Nile red was used to label nucleus and NPs, respectively. Cells were incubated with Nile red-loaded NPs (Nile red@NPs) (300 μg/mL) for 0.5, 1.5, 3.5, and 5.5 h, before incubation with Hoechst 33342 solution for 0.5 h. Afterward, the cells were rinsed with cold PBS to remove free Hoechst 33342 and Nile red@NPs samples. Finally, the stained cells were imaged by the CLSM system.

For flow cytometry analysis, the culture medium was replaced with Nile red@NPs and further incubated for 1, 2, 4, and 6 h, respectively. All the cells were washed with PBS and collected by trypsinization. After that, the cells were resuspended and analyzed by flow cytometry.

The exploration for endocytic inhibition was performed as follows. Cal-27 cells were pretreated with 0.1% NaN_3/50 mM 2-deoxyglucose (DOG) in serum-free DMEM for 1 h to suppress the energy-dependent endocytosis. Cells were pre-incubated in serum-free DMEM containing 5 mM methyl β-cyclodextrin (MβCD)

for 15 min at 37 ºC/5% CO_2 to inhibit MβCD. Cells were pretreated with the 225 mM sucrose in serum-free DMEM for 30 min at 37 ºC/5% CO_2 to inhibit the clathrin-mediated endocytosis. After exposure to the respective inhibitors and Nile red@NPs 2 h for all endocytic inhibition tests, the cells were washed with cold PBS and followed by quantifying the fluorescence intensity.

Intracellular free-radicals detection.

The intracellular generation of free radicals was monitored using H_2DCFH-DA fluorescent probe and recorded by the confocal laser scanning microscopy. In brief, Cal-27 cells were co-cultured with NPs and NMB@NPs (300 μg/mL) for 8 h at 37 °C. The cells were washed with PBS, subsequently incubated with H_2DCFH-DA (10 μM) for 30 min and stained with 1 mM Hoechst for 15 min at 37 °C in the dark. Afterward, the cells were irradiate with 808 nm laser irradiation (1 W/cm^2) for 10 min. The stained cells were imaged by the confocal laser scanning microscopy and quantified by flow cytometry.

***In vitro* analysis of cytotoxicity and apoptosis.**

Cal-27, HepG-2 cells (cancer cells) and LO2 cells (normal cells) were used to evaluate the cytotoxicity of NMB@NPs via MTT assays. Typically, Cal-27 cells were seeded in 96-well plates in 100 μL of complete DMEM medium and cultured overnight. The NMB@NPs suspensions with various concentrations were then added to these wells for 6 h. Cells were exposed to 808 nm laser irradiation at a power density of 1.0 W/cm^2 for 10 min and cultured for another 30 h. Cells unexposed group were taken as the control group. MTT reagent (in 20 μL PBS, 5 mg /mL) was added to each well and the plates then incubated for another 4 h. DMSO was used in each well (150 μL/well) instead of the medium and the plates were kept in the dark. The plate was gently agitated for 15 min before the light absorption value of each well at 570 nm was recorded by a microplate reader. HepG-2 and LO2 cells were conducted with the same method described above.

For live/death staining, Cal-27 cells were incubated with the dispersion of

NMB@NPs by the before mentioned NMB@NPs incubation and laser irradiation, and further incubated for another 30 h. After that, the cells stained with Calcein-AM/PI for 30 min in the cell-cultured container at the dark. Finally, the cell samples were imaged on confocal microscopy.

For flow cytometry analysis in double staining experiments, Cal-27 cells were cultured in dishes and incubated with the dispersion of NMB@NPs for 6 h and irradiated with a 808 nm laser (1.0 W/cm^2) for 10 min. After incubator for another 30 h, the cells were rinsed and collected by trypsinization. The precipitate after centrifugation precipitate was resuspended in PBS, stained with Annexin V-FITC/PI for 15 min, and finally analyzed by flow cytometry.

RNA-sequencing analysis.

The Cal-27 cells (1×10^6) were seeded in six-well plates and cultured for 24 h. Then different nanoparticles were added and incubated with cells for 12 h, and irradiated with 808 nm laser at 1 W/cm^2 for 10 min. Trizol was added to extract RNA after continued culture for another 12 h. BGISEQ-500 was applied in RNA sequencing. RSEM was used for transcription levels quantification. When the Q value was less than or equal to 0.001 and the fold change was greater than or equal to 2, a differentially expressed gene (DEG) was identified. Ggplot2 was employed to generate the volcano graphs. Pheatmap was employed to generate the heat maps. Phyper was utilized to complete Kyoto encyclopedia of genes and genomes (KEGG) pathway enrichment analysis. When the q-values were less than or equal to 0.05, a significant enrichment was identified. Cytoscape was used for the protein–protein interaction network generation.

Animal model.

After surgical excision of a tumor from the oral, a tumor tissue sample was cut into ≈3 mm pieces and then implanted into the female BALB/c nude mice (5~6 weeks old, ~15 g). Tumors start to grow in the area of implantation after 1-2 months. When the tumor reached a volume of 1000 mm^3, the mice were euthanized and the

xenografts were subsequently transplanted into another group of mice use the same protocol. In this work, after the third generation of animals (The generations of the patient-derived material numbered P3), the tumor tissues were implanted into the female BALB/c nude mice (8~9 weeks old, ~15 g) and was used for *in vivo* experiments. All animal experiments were performed with the permission of the Animal Ethics Committee，according to the guidelines approved by Guangdong Administration of Experimental Animals and Shanghai Jiao Tong University School of Medicine (Approved number: HN-2543, HN-2539).

Tumor fluorescence imaging and photothermal imaging.

Oral tumor xenografted nude mice were treated with intravenous injection via tail vein with NMB@NPs at a dose of 2 mg/kg (NMB). The NIR-II fluorescence images was taken 0-24 h after injection using the small animal imaging system equipped with a 1100 nm longpass filter (1100 LP) and 808 nm laser source. To evaluate the tissue distributions of NMB@NPs, the mice were sacrificed to collected the major organs and tumors at 24 h postinjection for *ex vivo* fluorescence imaging. For photothermal imaging, mice were randomly divided into three groups and injected with 200 μL of PBS, NPs, and NMB@NPs via the tail vein. Tumors of all groups were treated under 808 irradiation (1 W/cm^2) for 10 min after 20 h post-injection. Meanwhile, the real-time temperature of the tumors and the photothermal images of the mice were recorded using an infrared thermal camera.

***In vivo* cancer inhibition efficacy.**

The oral tumor-bearing Balb/c mice at an initial tumor size of 50–60 mm^3 were randomized into six groups (n=5, each group). The treatment scheme was as follows: (1) PBS (-Laser), (2) PBS (+Laser), (3) NPs (-Laser), (4) NPs (+Laser), (5) NMB@NPs (-Laser), (5) NMB@NPs (+Laser). NMB@NPs at a NMB dose of 2 mg NMB $(kg)^{-1}$ body weight and a NPs dose of 20 mg/kg body weight. Then, the tumors were suffered from 808 nm light exposure at 1.0 W/cm^2 for 10 min after intreating with PBS, NPs and NMB@NPs, respectively. The tumor volumes and

body weights were monitored every 3 days. The tumor volume was estimated using the formula: tumor volume = length×(width)^2/2, Relative tumor volume was expressed as V/V_0 (V_0 is the initial mouse volume). A total of 15 days later, major organs as well as tumors were collected and sectioned for H&E staining.

References

[1] H. Xiang, H. Lin, L. Yu, Y. Chen, *ACS Nano* **2019**, *13*, 2223.

[2] W. Xiao, P. Wang, C. Ou, X. Huang, Y. Tang, M. Wu, W. Si, J. Shao, W. Huang, X. Dong, *Biomaterials* **2018**, *183*, 1.

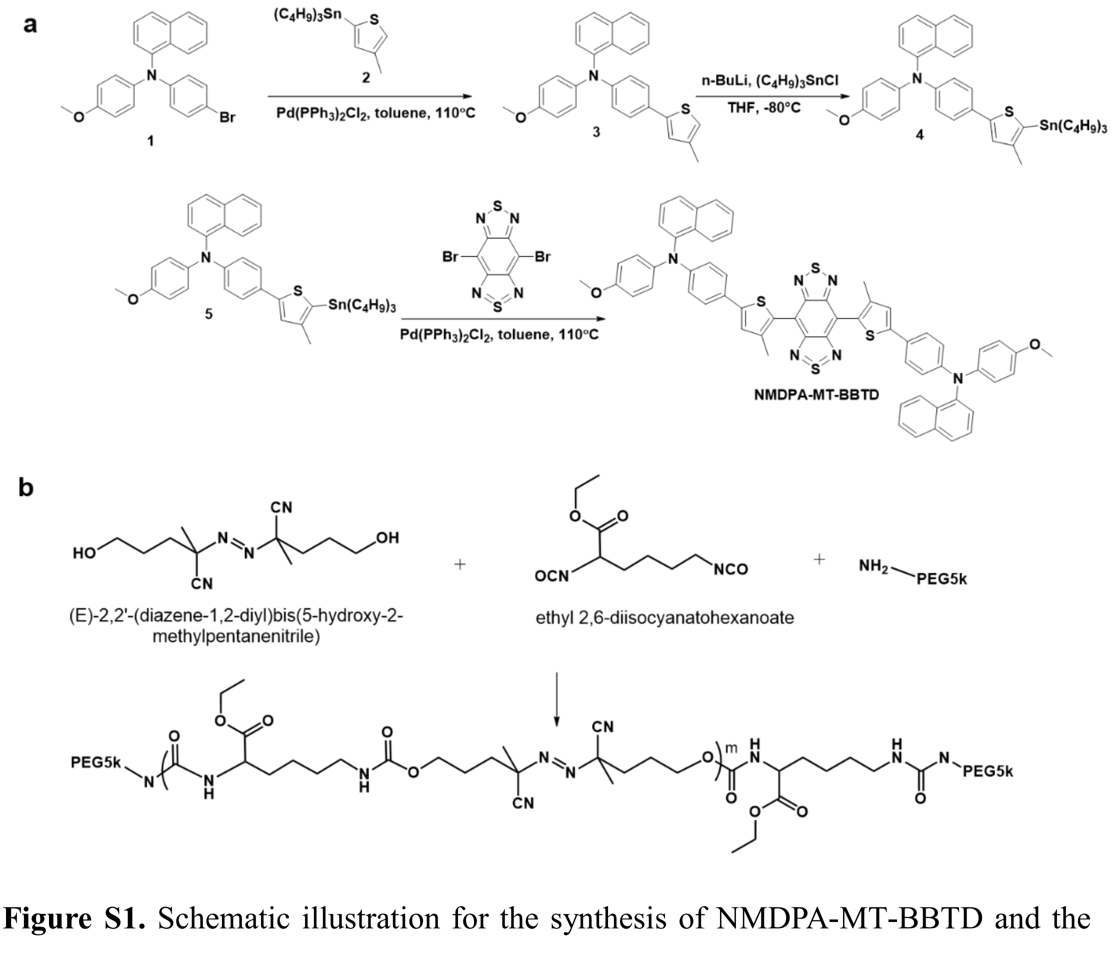

Figure S1. Schematic illustration for the synthesis of NMDPA-MT-BBTD and the thermolabile polymer.

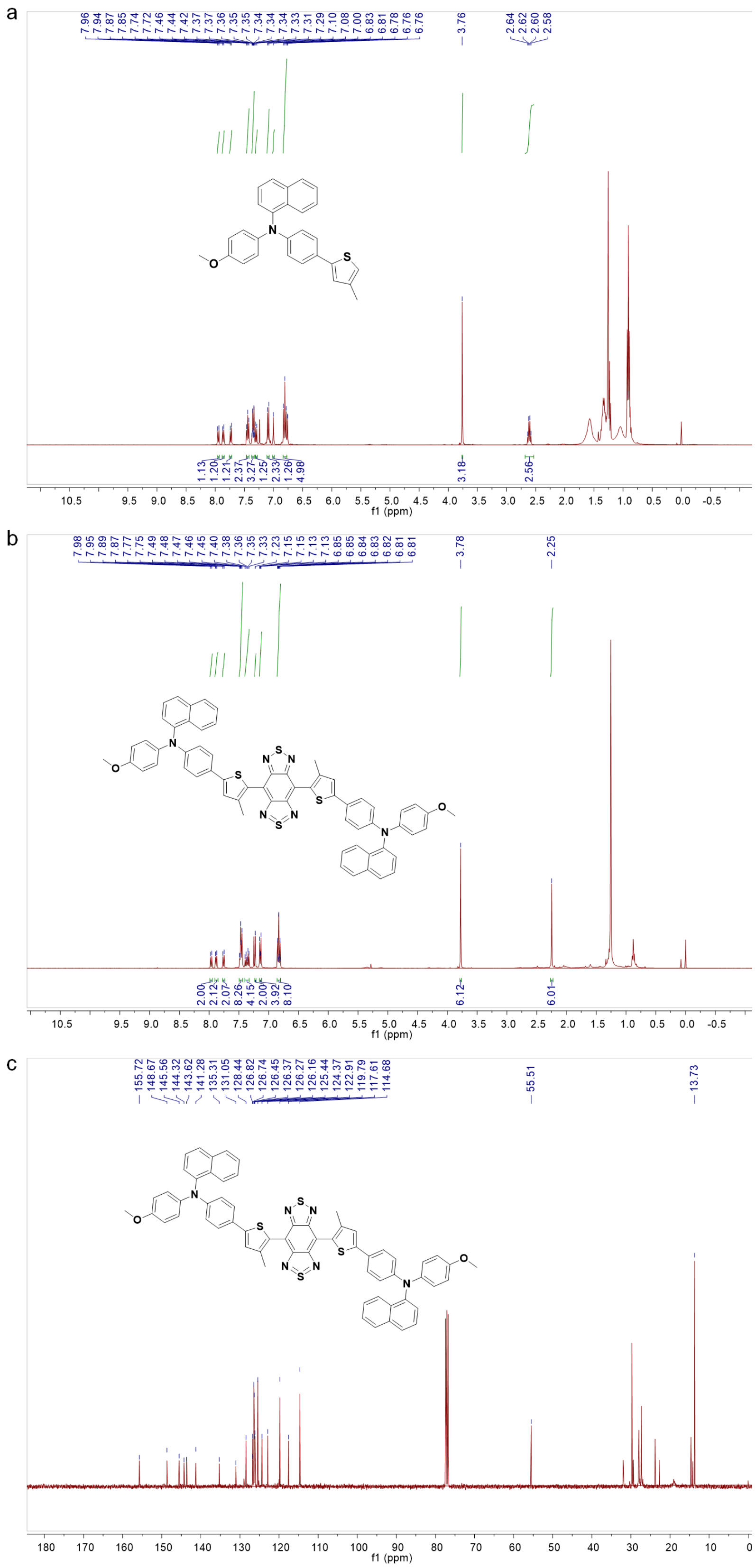

Figure S2. Characterization of compound 3 and NMDPA-MT-BBTD by NMR. (a) ^{1}H NMR spectrum of compound 3. (b) ^{1}H NMR spectrum of NMDPA-MT-BBTD. (c) ^{13}C NMR spectrum of NMDPA-MT-BBTD.

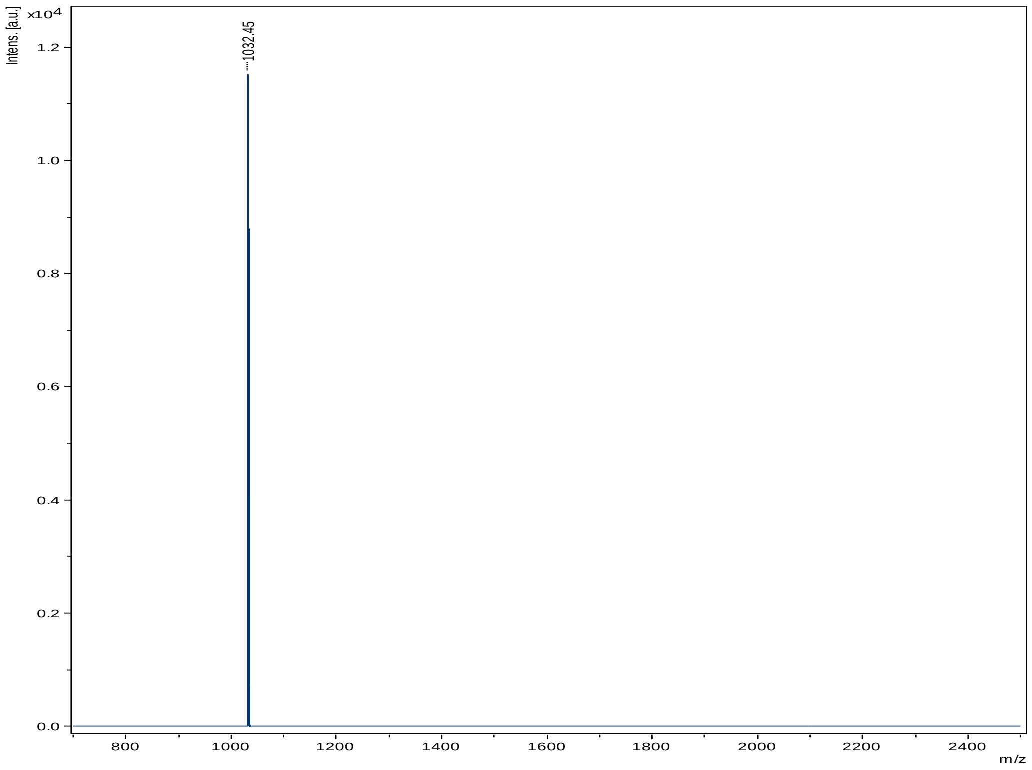

Figure S3. MALDI-TOF spectrum of NMDPA-MT-BBTD.

Table S1. Cartesian coordinates of optimized NMDPA-MT-BBTD calculated by the DFT, B3LYP/6-31G (d, p), Gaussian 09 program.

Atom	x	y	z
C	-13.3345	-2.65114	0.76214
C	-12.5422	-1.53831	0.40432
C	-11.3665	-1.25561	1.07447
C	-10.9419	-2.08784	2.1633
C	-11.7432	-3.229	2.50288
C	-12.939	-3.4865	1.78086
N	-10.5951	-0.1101	0.69877
C	-9.31166	-0.26741	0.13905
C	-11.2446	1.16155	0.71103
C	-11.2439	1.99632	-0.41989
C	-11.8919	3.22313	-0.39322
C	-12.5785	3.64008	0.75687
C	-12.5974	2.81004	1.88338
C	-11.9246	1.58717	1.85593
C	-8.36705	0.77658	0.18774
C	-7.0978	0.61051	-0.3474
C	-6.69677	-0.60306	-0.93857
C	-7.64449	-1.64334	-0.97189
C	-8.92364	-1.48141	-0.45815
O	-13.1905	4.85859	0.67468
C	-13.89	5.3339	1.81333
C	-5.35674	-0.79439	-1.49094
C	-4.94546	-1.69899	-2.44836
C	-3.56363	-1.64751	-2.76921
C	-2.88984	-0.67379	-2.0384
S	-4.00452	0.17761	-0.9721
C	-1.47464	-0.34109	-2.03157
C	-0.98475	0.99213	-1.99689
C	0.45021	1.32556	-1.99265
C	1.47456	0.34157	-2.03146
C	0.98467	-0.99164	-1.99697
C	-0.45029	-1.32506	-1.99285
N	1.77313	-2.08539	-1.95231
S	0.78567	-3.37783	-1.87465

N	-0.67311	-2.65393	-1.90823
N	0.67302	2.6544	-1.90781
N	-1.77322	2.08586	-1.95212
C	13.33459	2.65085	0.76211
C	12.54216	1.53812	0.40421
C	11.3666	1.25536	1.07448
C	10.9422	2.08741	2.16351
C	11.74362	3.22847	2.50319
C	12.93928	3.48604	1.78103
N	10.59512	0.10995	0.6987
C	9.31162	0.26739	0.13909
C	11.24446	-1.16176	0.71081
C	11.24378	-1.99635	-0.42023
C	11.89176	-3.2232	-0.39372
C	12.57821	-3.64037	0.75635
C	12.59715	-2.8105	1.88298
C	11.92442	-1.58758	1.85568
C	8.367	-0.77659	0.18762
C	7.09773	-0.6104	-0.34744
C	6.69669	0.60329	-0.93835
C	7.64441	1.64357	-0.97148
C	8.92358	1.48153	-0.45783
O	13.19013	-4.85891	0.67399
C	13.88963	-5.33446	1.81261
C	5.35666	0.79474	-1.49068
C	4.9454	1.69953	-2.44793
C	3.56358	1.64813	-2.76881
C	2.88976	0.67427	-2.0382
S	4.00443	-0.17737	-0.97207
S	-0.78577	3.37829	-1.87421
C	-9.77725	-1.82076	2.93262
C	-9.41113	-2.64875	3.97017
C	-10.1883	-3.78672	4.2914
C	-11.3287	-4.06534	3.57467
C	9.77763	1.82026	2.93295
C	9.41169	2.64808	3.9707
C	10.18893	3.78594	4.29203
C	11.32933	4.06463	3.57519

C	-2.97224	-2.50992	-3.85197
C	2.97227	2.51073	-3.85146
H	-14.2514	-2.84888	0.21505
H	-12.8514	-0.88953	-0.40885
H	-13.5394	-4.35035	2.05268
H	-10.7273	1.67863	-1.31958
H	-11.8938	3.87306	-1.262
H	-13.1168	3.10509	2.78723
H	-11.9321	0.95171	2.73563
H	-8.6361	1.72185	0.64526
H	-6.40262	1.44421	-0.30971
H	-7.36991	-2.60294	-1.39836
H	-9.62865	-2.30327	-0.50812
H	-14.2927	6.30875	1.53472
H	-14.7179	4.66775	2.08887
H	-13.2243	5.45287	2.67794
H	-5.63267	-2.36885	-2.9538
H	14.25141	2.84865	0.21492
H	12.85122	0.88947	-0.40912
H	13.53974	4.34981	2.05293
H	10.72724	-1.6785	-1.31989
H	11.89367	-3.87301	-1.2626
H	13.11643	-3.10572	2.78682
H	11.93185	-0.95226	2.73548
H	8.63606	-1.72195	0.64495
H	6.40254	-1.4441	-0.30987
H	7.36981	2.60327	-1.39771
H	9.62857	2.30341	-0.50763
H	14.29223	-6.30929	1.53385
H	14.71752	-4.66842	2.08831
H	13.22384	-5.45353	2.67715
H	5.63263	2.36949	-2.95322
H	-9.17611	-0.95113	2.6947
H	-8.51895	-2.42657	4.54807
H	-9.88445	-4.43454	5.10836
H	-11.9371	-4.93149	3.82167
H	9.17641	0.95072	2.69493
H	8.51959	2.42584	4.54867

H	9.88528	4.43363	5.10915
H	11.93774	4.93071	3.82227
H	-3.71118	-2.68029	-4.64146
H	-2.09306	-2.04649	-4.30499
H	-2.65978	-3.48273	-3.46058
H	3.71126	2.68124	-4.64086
H	2.09311	2.04739	-4.30463
H	2.65978	3.48348	-3.45991

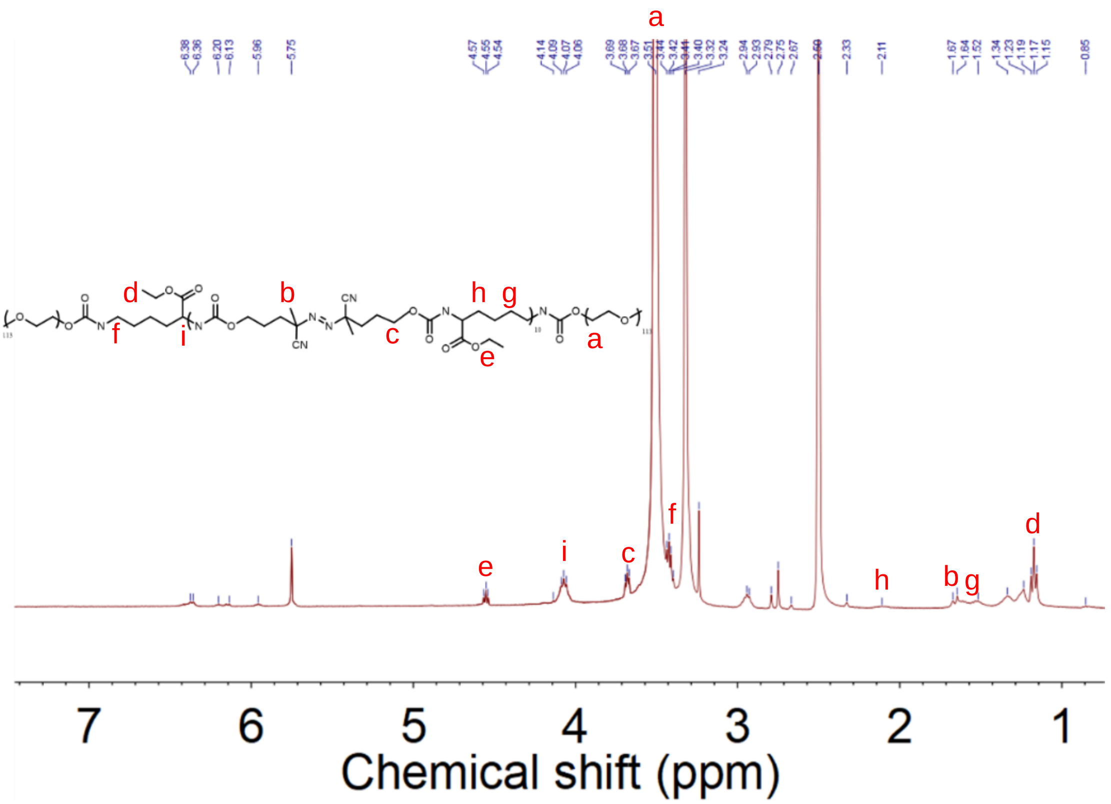

Figure S4. ^{1}H NMR spectrum recorded for the thermolabile polymer in DMSO-d_6.

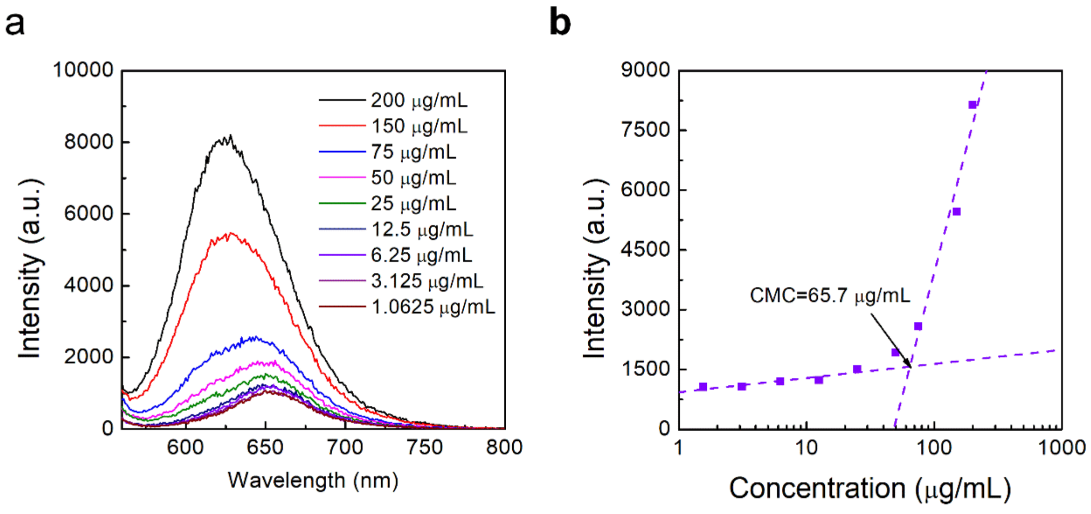

Figure S5. Determination of critical micelle concentration (CMC) of NPs by a Nile red (Nile red@NPs) assay. (a) The fluorescence emission spectrum of Nile red@NPs at various mass ratios. (b) The fluorescence intensity of Nile red@NPs at 650 nm was plotted against the polymer concentrations for CMC determination (CMC=65.7 μg/mL).

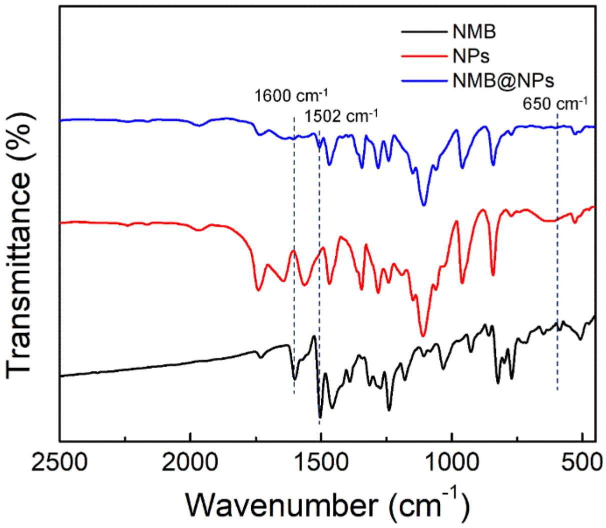

Figure S6. FT-IR spectra of NMB, thermoliable polymer NPs, and NMB@NPs, respectively.

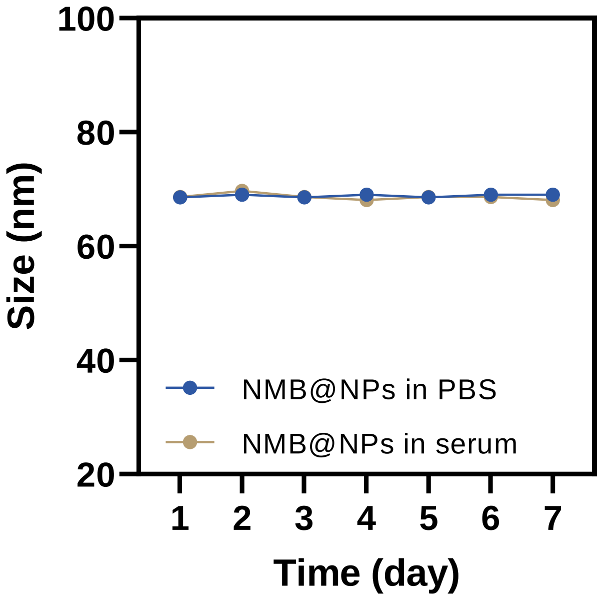

Figure S7. *In vitro* stability evaluation for NMB@NPs upon dispersing in PBS solution and serum-containing medium at 37 ^{o}C for different durations.

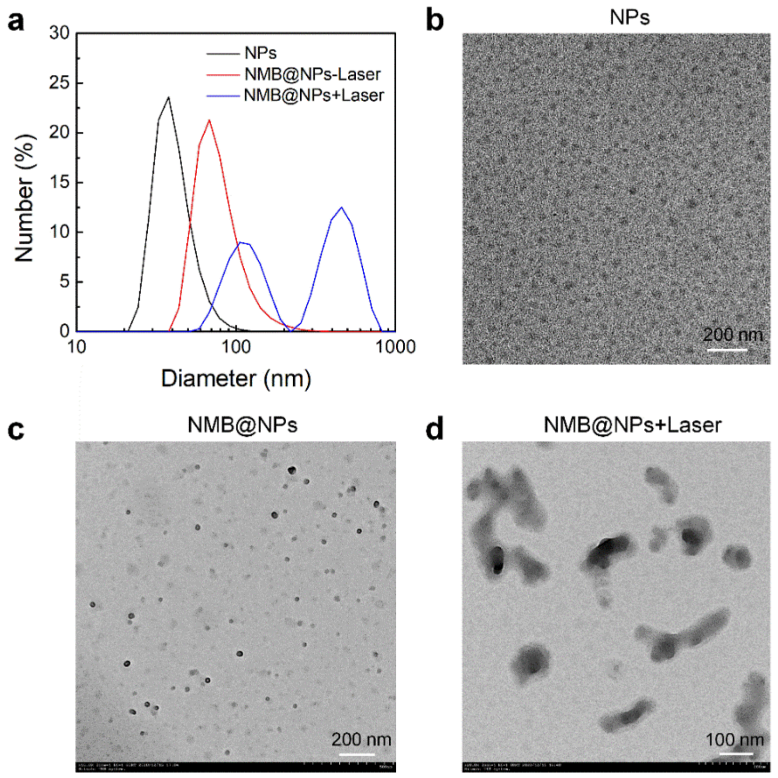

Figure S8. Characterization of NPs and NMB@NPs. (a) Size distribution of NPs and NMB@NPs measured by DLS. TEM image of (b) NPs, (c) NMB@NPs, and (d) MB@NPs under 808 nm laser irradiation.

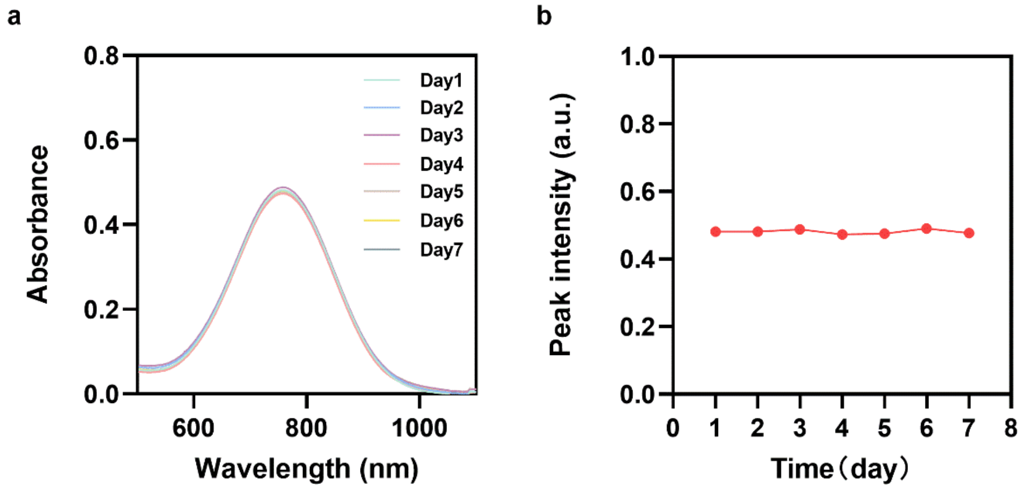

Figure S9. *In vitro* stability evaluated for the NMB@NPs aqueous dispersion upon storing in different durations. (a) UV-vis spectra of NMB@NPs, and (b) The maximum peak intensity obtained in (a).

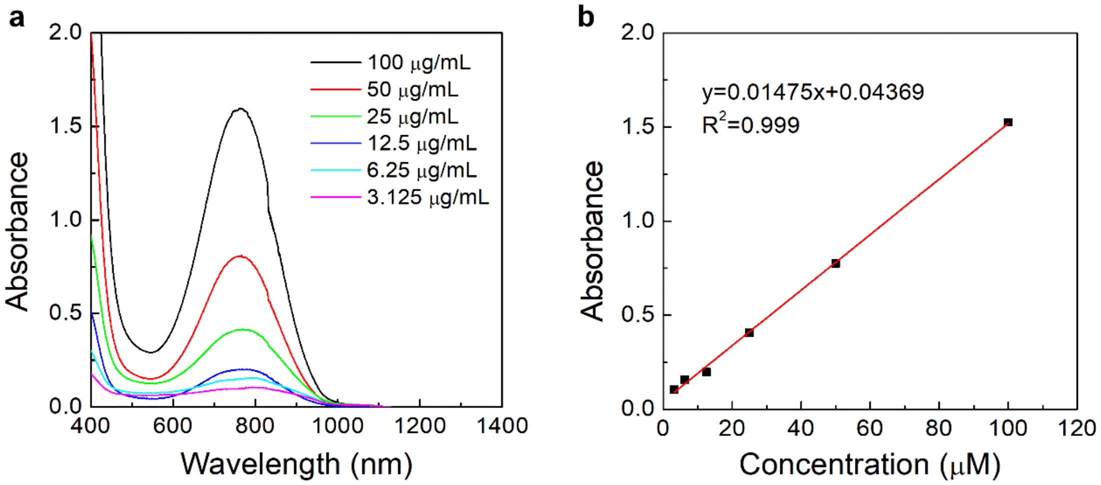

Figure S10. Characterization of NMB. (a) The absorption spectroscopy according to the concentration of NMB. (b) Standard curve plotted through measuring absorbance intensity of UV-vis spectra under different concentration of NMB.

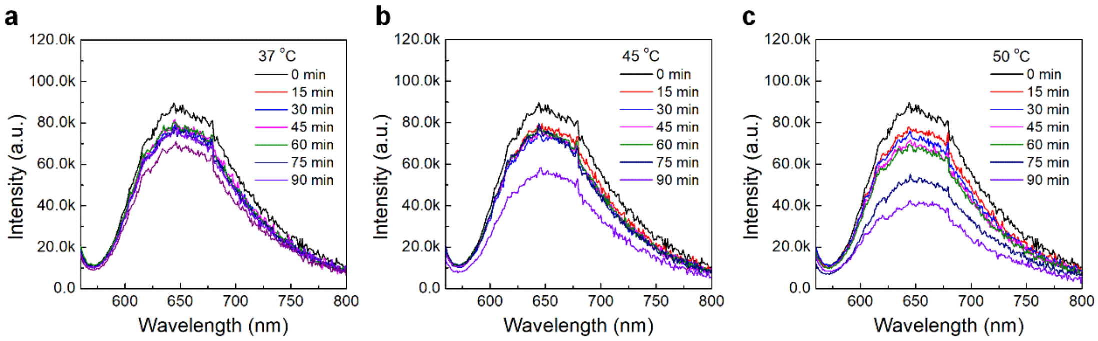

Figure S11. Degradation of Nile red@NPs under different temperature conditions. The fluorescence emission spectra of Nile red@NPs measured per 15 minutes during a period of 90 minutes at (a) 37 °C, (b) 45 °C, and (c) 50 °C.

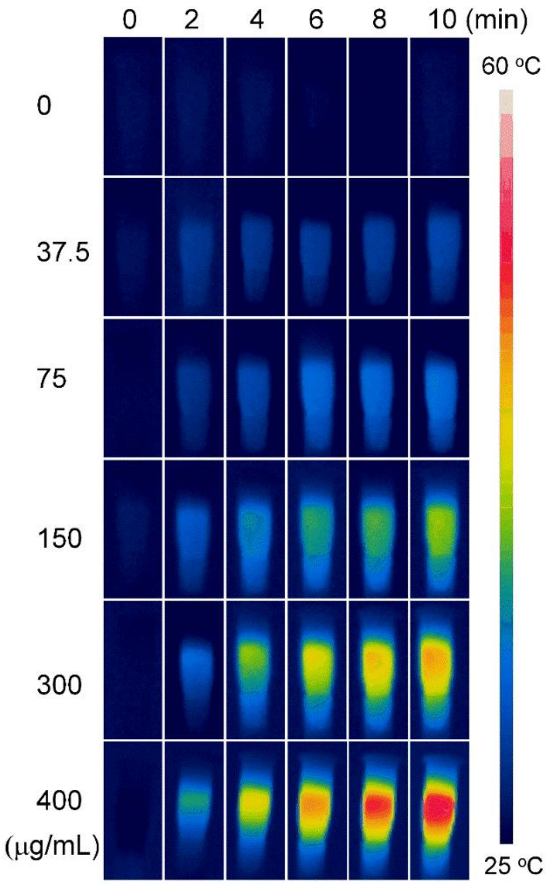

Figure S12. Time-dependent temperature images of NMB@NPs at various concentrations upon 808 nm laser (1.0 W/cm^2) for 10 min.

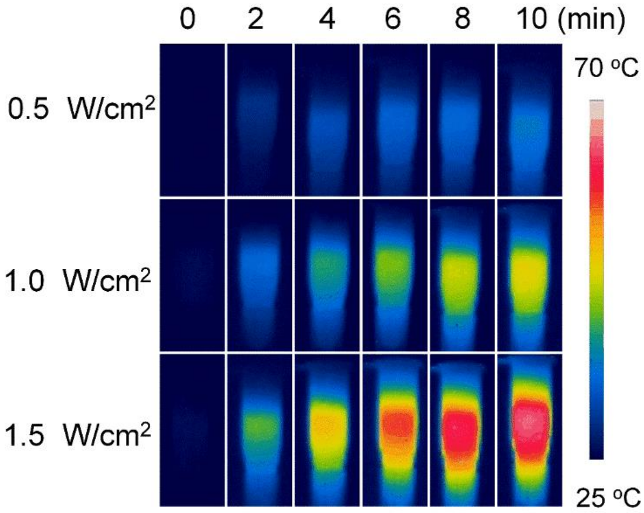

Figure S13. Time-dependent temperature images of NMB@NPs (0.3 mg/mL) obtained after 10 minutes of irradiation by a 808 nm laser at different power density.

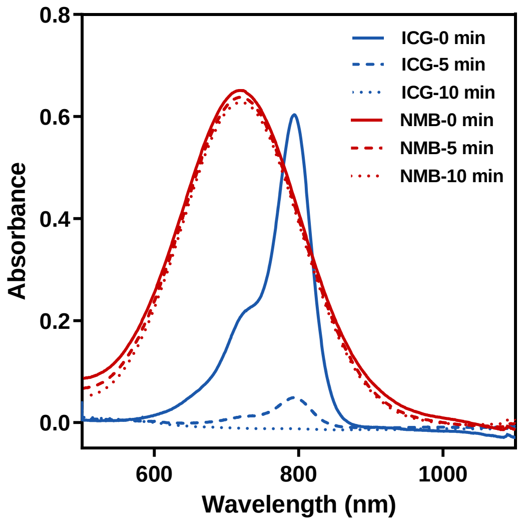

Figure S14. The photostability of NMB and ICG was examined against laser irradiation at 808 nm, 1 W/cm^2.

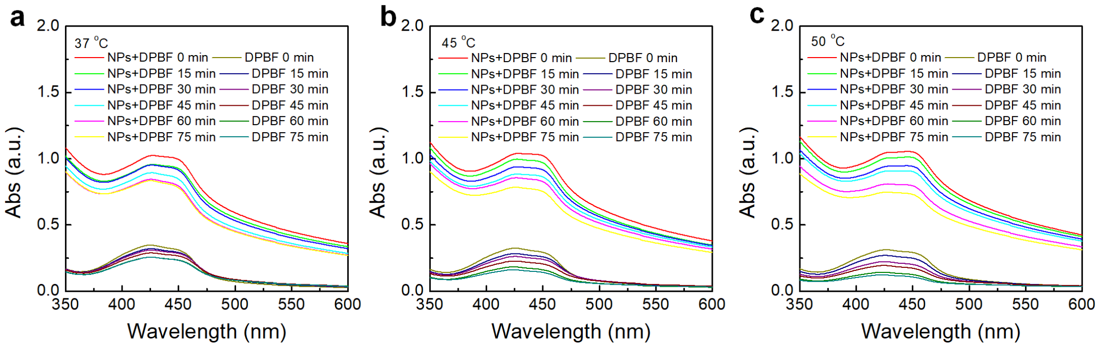

Figure S15. *In vitro* detection of free radicals generated from NPs by DPBF in DMF at different time points at (a) 37 °C, (b) 45 °C, and (c) 50 °C, respectively.

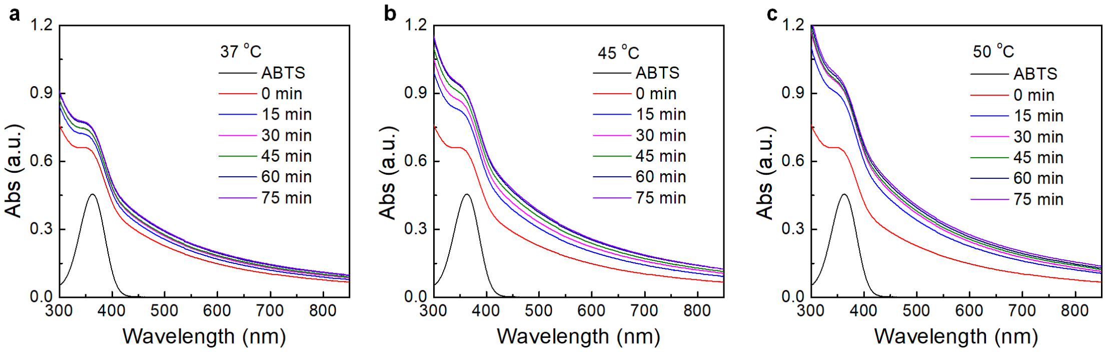

Figure S16. *In vitro* detection of free radicals generated from NPs by ABTS at different time points at (a) 37 °C, (b) 45 °C, and (c) 50 °C, respectively.

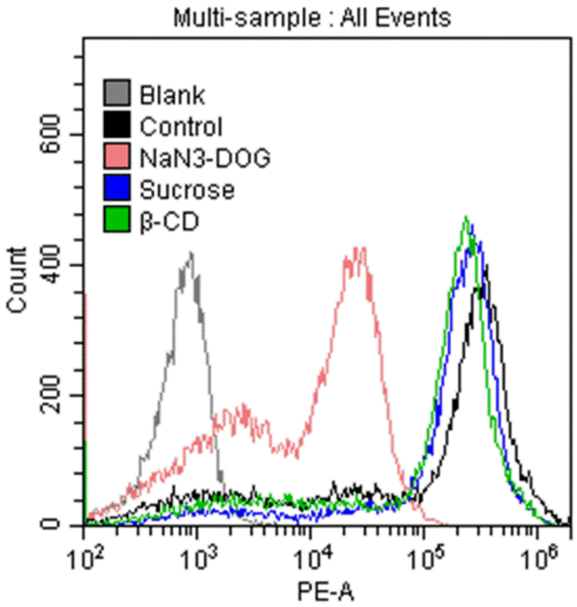

Figure S17. Flow cytometry analysis of Cal-27 cells towards Nile red@NPs (300 μg mL^{-1}) in the presence of diverse endocytosis inhibitors.

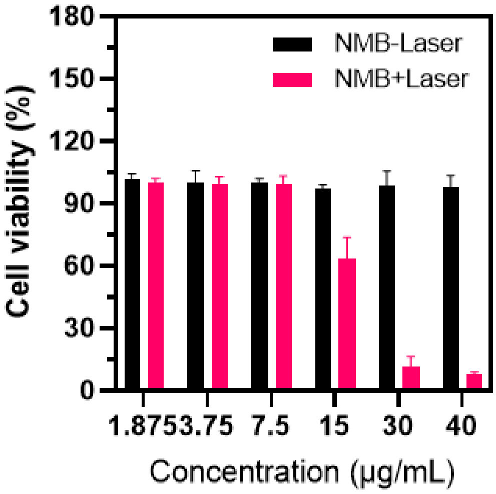

Figure S18. Cell viability of Cal-27 cells after incubating with NMB before and after irradiation.

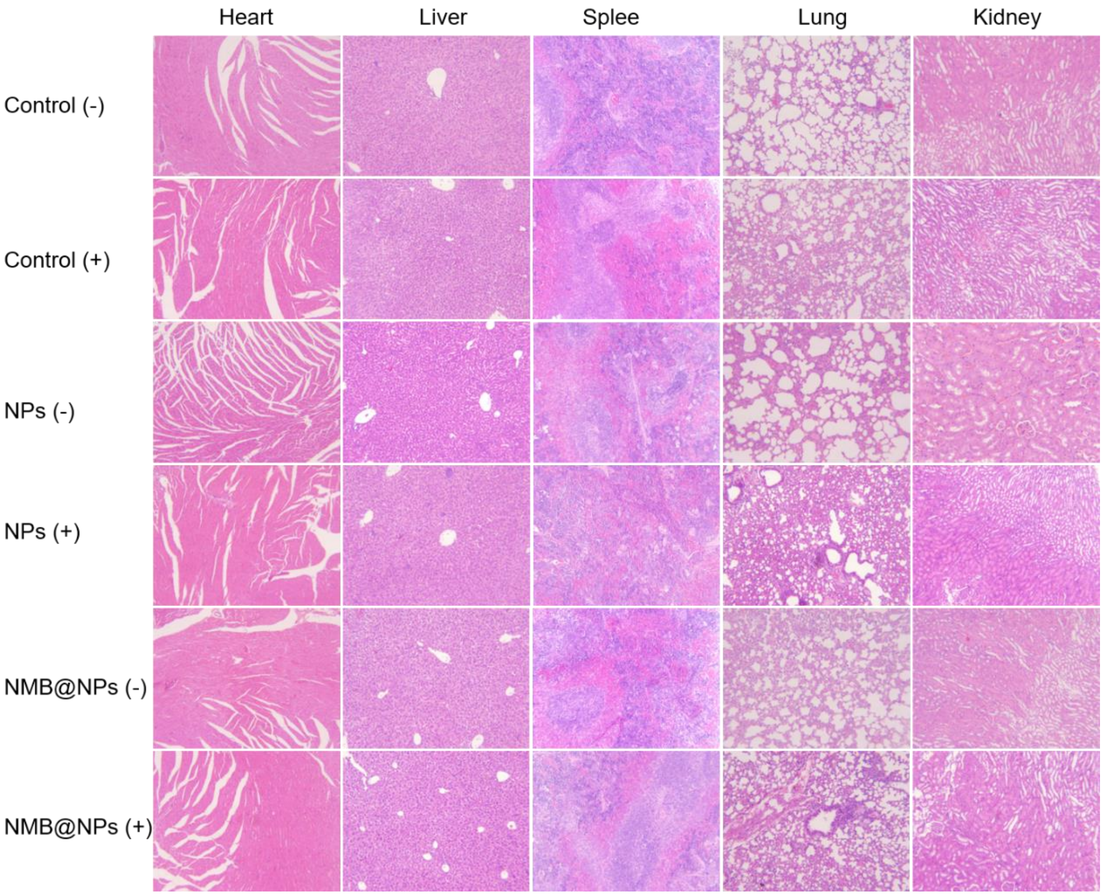

Figure S19. Representative H&E (20x) stained images of main organs (heart, liver, spleen, lung, and kidney) of mice upon different treatments.

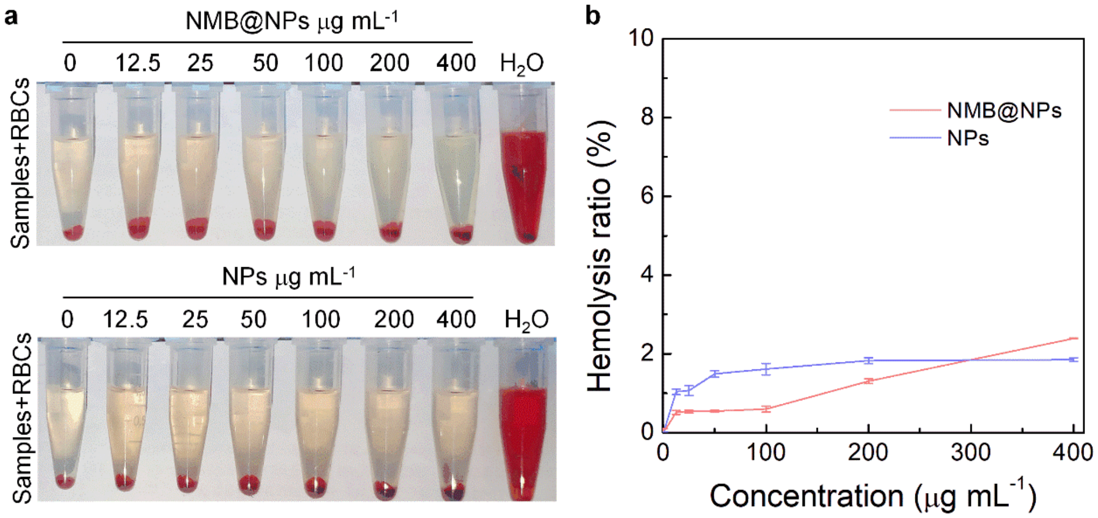

Figure S20. Hemolysis assay of NMB@NPs at different concentrations. (a) Photographic images of hemolysis. (b) Concentration-dependent hemolysis quantification of NMB@NPs and NPs.

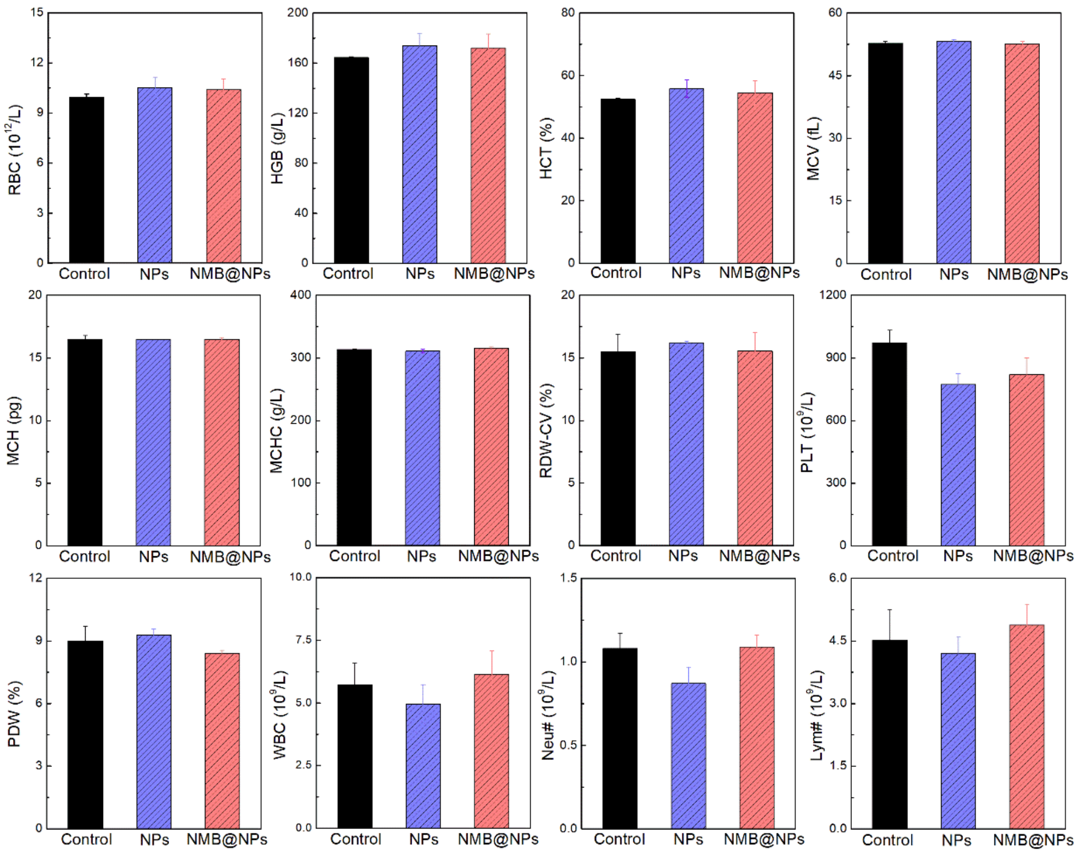

Figure S21. Blood hematology analyses of mice. No significant difference in all blood test data. Error bars represent standard deviation, n = 3.

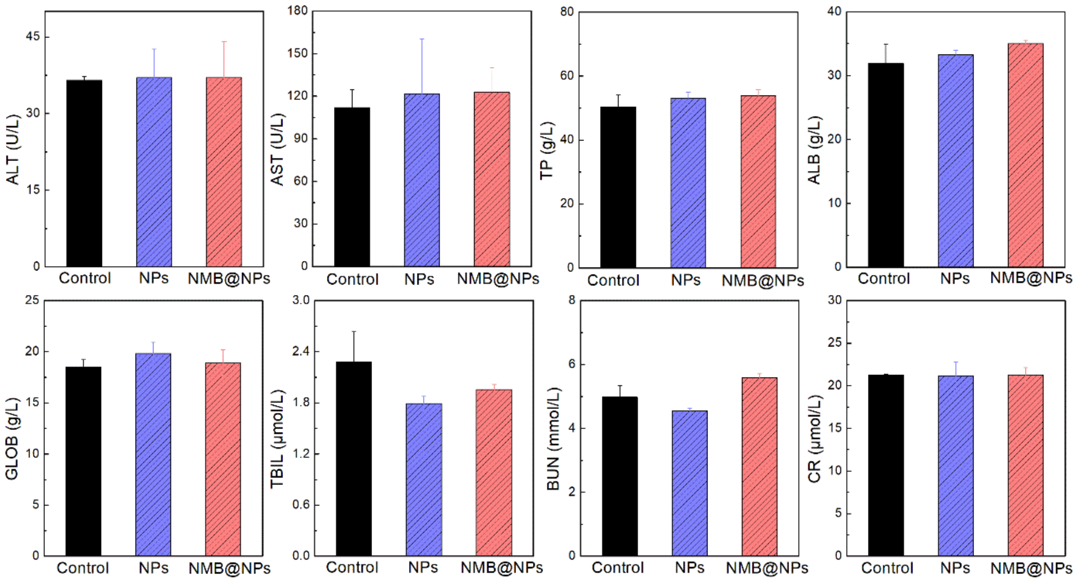

Figure S22. Blood biochemistry analyses of mice. Error bars represent standard deviation for n = 3.
